# Effect of Briefing on Acupuncture Treatment Outcome Expectations, Pain, and Adverse Side Effects Among Patients With Chronic Low Back Pain: A Randomized Clinical Trial

**DOI:** 10.1001/jamanetworkopen.2021.21418

**Published:** 2021-09-10

**Authors:** Jürgen Barth, Stefanie Muff, Alexandra Kern, Anja Zieger, Stefanie Keiser, Marco Zoller, Thomas Rosemann, Benno Brinkhaus, Leonhard Held, Claudia M. Witt

**Affiliations:** 1 Institute for Complementary and Integrative Medicine, University Hospital Zurich and University of Zurich, Zurich, Switzerland; 2 Department of Biostatistics at the Epidemiology, Biostatistics and Prevention Institute, University of Zurich, Zurich, Switzerland; 3 Department of Mathematical Sciences, Norwegian University of Science and Technology, Trondheim, Norway; 4 Institute of Primary Care, University Hospital Zurich, Zurich, Switzerland; 5 Charité–Universitätsmedizin Berlin, corporate member of Freie Universität Berlin and Humboldt-Universität zu Berlin, Institute of Social Medicine, Epidemiology and Health Economics, Berlin, Germany; 6 Center for Integrative Medicine, University of Maryland, School of Medicine, Baltimore

## Abstract

**Importance:**

In observational studies, patients’ treatment outcome expectations have been associated with better outcomes (ie, a placebo response), whereas concerns about adverse side effects have been associated with an in increase in the negative effects of treatments (ie, a nocebo response). Some randomized trials have suggested that communication from clinicians could affect the treatment outcomes by changing patients’ expectations.

**Objective:**

To investigate whether treatment outcome expectations and reported adverse side effects could be affected by different briefing contents before a minimal acupuncture treatment in patients with chronic low back pain (CLBP).

**Design, Setting, and Participants:**

This randomized single-blinded clinical trial was conducted among patients with CLBP at 1 outpatient clinic in Switzerland who had a pain intensity of at least 4 on a numeric rating scale from 0 to 10. Different recruitment channels were used to enroll patients. Data were collected from May 2016 to December 2017 and were analyzed from June to November 2018.

**Interventions:**

Patients were randomized to receive either a regular expectation briefing or a high expectation briefing (effectiveness) and either a regular adverse side effect briefing or an intense adverse side effect briefing (adverse side effect) in a 2 × 2 factorial design. The intervention (briefing sessions and written materials) was standardized and delivered before the acupuncture treatment, with additional booster informative emails provided during the 4-week, 8-session acupuncture course.

**Main Outcomes and Measures:**

The primary end point was the patients’ expectations regarding the effectiveness of the acupuncture treatment (Expectation for Treatment Scale [ETS]) after the briefing and the subsequent pain intensity (numeric rating scale). The primary end point for the adverse side effect briefing was the adverse side effect score at the end of the acupuncture treatment, derived from session-by-session assessments of adverse side effects.

**Results:**

A total of 152 patients with CLBP (mean [SD] age, 39.54 [12.52] years; 100 [65.8%] women) were included. The estimated group difference (regular vs high) for the ETS was −0.16 (95% CI −0.81 to 0.50, *P* = .64), indicating no evidence for a difference between intervention groups. There was also no evidence for a difference in pain intensity at the end of the acupuncture treatment between the groups with different expectation briefings. The adverse side effects score in the group with the intense adverse side effect briefing were estimated to be 1.31 times higher (95% CI, 0.94 to 1.82; *P* = .11) than after a regular adverse side effect briefing, but the finding was not statistically significant.

**Conclusions and Relevance:**

In this study, suggestions regarding treatment benefits (placebo) and adverse side effects (nocebo) did not affect treatment expectations or adverse side effects. Information regarding adverse side effects might require more research to understand nocebo responses.

**Trial Registration:**

German Clinical Trials Register Identifier: DRKS00010191

## Key Points

**Question:**

Can briefing suggestions before an acupuncture treatment in patients with chronic low back pain (CLBP) influence outcome expectations and reported adverse side effects?

**Findings:**

This randomized clinical trial of 152 patients with CLPB found that briefings about treatment benefits did not significantly change outcome expectations of effectiveness, pain, or adverse side effects during an acupuncture treatment.

**Meaning:**

In this study, there was no statistically significant effect of suggestions about treatment benefits or adverse side effects on patients’ treatment expectations or experience of adverse side effects.

## Teaser

This randomized clinical trial investigates whether different briefing contents before a minimal acupuncture treatment among patients with chronic low back pain affect treatment outcome expectations and reported adverse side effects.

## Introduction

The placebo response to medical treatments is increasingly recognized in clinical practice,^1,2^ and nocebo effects are highly prevalent in many treatments.^3^ Experimental studies have shown that positive and negative suggestions can elicit placebo or nocebo effects,^4,5^ but physicians also regularly use positive suggestions during consultations.^6^ Given that physicians also refer patients to treatments, such as acupuncture, they inform their patients about risks and benefits. Within this communication, clinicians aim to increase placebo effects and minimize nocebo effects.^7,8^

Positive messages may improve treatment outcomes^9,10^; therefore, studies have investigated pretreatment briefings and specific communication styles. It was shown that additional communication about potential benefits in patients undergoing heart surgery had a positive impact on their disability compared with usual care, but it was not more effective than an overall supportive communication of a similar length.^11^ Communicating the harms of a treatment might increase adverse side effect reporting^12^; however, this needs more research. Studies using an additional pretreatment safety briefing are still in progress.^13^ Studies on the adverse side effects of medication have shown mixed results regarding the impact of the initial information on adverse side effects and adverse side effect reporting afterwards.^14^

In this study, we used acupuncture as a research model for a nonpharmacological intervention because this intervention is widely available in routine care for patients with chronic low back pain (CLBP). Physicians can inform patients about the acupuncture benefits from 2 different perspectives: focusing either on overall effectiveness (acupuncture compared with usual care) or acupuncture-specific effects (acupuncture compared with sham acupuncture). This research model is particularly interesting because acupuncture was found to be statistically significantly and clinically relevantly more effective than usual care (standardized mean difference [SMD], 0.52).^15^ However, compared with sham acupuncture, the association was statistically significant but not clinically relevant (SMD, 0.30).^15^ These 2 perspectives resulted in different recommendations in clinical practice guidelines,^16,17^ followed by heterogeneous advice that physicians could provide to patients when referring to acupuncture treatment.

Acupuncture studies have shown that patients with high expectations can have better outcomes than patients with lower expectations.^18,19,20,21,22^ Acupuncture has served before as a suitable placebo research model,^23,24^ and superficial needling of nonacupuncture points (ie, minimal acupuncture) was more effective than no acupuncture in a randomized clinical trial.^25^

This study aimed to investigate the impact of 2 verbal briefing interventions on patients’ treatment expectations before minimal acupuncture treatment, the experience of adverse side effects during treatment, and pain reduction after treatment. The verbal briefings about acupuncture had different contents regarding effectiveness (regular vs high expectation briefings) and adverse side effects (regular vs intense expectation briefing) of the acupuncture.

## Methods

### Study Design

We performed a randomized single-blinded 4-armed trial with a 2 × 2 factorial design with 2 independent factors (effectiveness briefing and adverse side effect briefing plus booster emails). All patients received a standardized 4-week minimal acupuncture treatment (8 sessions). The standardized briefing intervention with the physician took place before the first acupuncture session (lasting approximately 30 minutes) and before the third acupuncture session (lasting approximately 15 minutes). The trial was conducted in accordance with the Declaration of Helsinki^26^ and the International Conference on the Harmonisation of Good Clinical Practice. It was approved by the cantonal ethics committee of Zurich. All participants gave written informed consent. This report follows the Consolidated Standards of Reporting Trials (CONSORT) reporting guideline.^27^ The study protocol and the statistical analysis plan are available in Supplement 1.

The sample size calculation showed that the study needed 128 patients to have 80% power (2-sided test; α = .05) to show a clinically meaningful effect with an SMD of 0.5 on the patients’ treatment expectations. Taking into account dropouts, 150 patients were planned (eAppendix 1 in Supplement 2).

### Participants

We included patients aged 18 to 65 years with clinically diagnosed CLBP^28^ with an average pain intensity of at least 4 on a numeric rating scale (NRS) from 0 to 10 and pain at least half of the days per week in the last 6 months. Patients with an acupuncture treatment within the last 12 months were excluded (eAppendix 2 in Supplement 2).

Study participants were recruited via email newsletters from among the staff of the University Hospital Zurich and by flyers or emails directed at university institutions, fitness gyms, practices of physicians, and via the institutes’ website (first patient was recruited May 5, 2016; last patient completed the study on December 6, 2017). Interested persons could contact the study center and participate in prescreening via telephone followed by a face-to-face consultation with a physician to assess their final eligibility.

### Briefing Interventions

The intervention (2 oral briefing sessions and written materials) was standardized and delivered by a single study physician (M.Z.) before the treatment, followed by 2 booster emails sent after acupuncture sessions 3 and 6. The briefing content used evidence-based information about the effectiveness and adverse side effects of acupuncture for CLBP; however, the groups differed in the emphasized aspects of the information (eg, positive framing in the high expectation briefings) and the details provided (eAppendix 3 in Supplement 2). All patients were informed that they received an acupuncture treatment that has been shown to be beneficial for CLBP. In the high expectation group, emphasis was placed on the clinically relevant difference between acupuncture and usual care (responder rates, 48% vs 27%) and, in the low expectation group, on the small difference between acupuncture and sham acupuncture (responder rates, 48% vs 44%) based on the findings of an earlier study.^29^

The briefing about adverse side effects was different between the regular and intense adverse side effect communication. In the regular adverse side effect briefing group, the patients were simply given the study information. In the intense adverse side effect briefing group, patients additionally received a brochure that was discussed in detail with the study physician. The physician marked typical adverse side effects (eg, bleeding, hematoma, pain) mentioned in the brochure to emphasize them.

### Acupuncture Treatment

All patients received the same standardized minimal acupuncture for free (8 sessions, 2 times per week for 45 minutes), which has been systematically developed^25,27,28,29,30^ and used in a CLBP trial before and was more effective than no treatment (eAppendix 4 and eAppendix 5 in Supplement 2). The treatment was delivered by 3 specially trained treatment practitioners. If required, patients could use nonsteroidal anti-inflammatory drugs but were asked to document this in a medication diary.

### Procedures

We performed central block randomization (variable block length in a 1:1:1:1 ratio, stratified by sex) with secuTrial software (Clinical Trial Center, University Hospital Zurich). The sequence was generated with R version 3.1.0 (R Project for Statistical Computing) by a statistician not further involved in the study.

Before and during the study, patients were not made aware of the study aim of changing expectations by using different verbal briefings. They were informed that the purpose of the study was to investigate the impact of expectations on treatment effects in an observational acupuncture study. After the end of the study, patients were fully informed and asked to provide a posttreatment guess to measure whether the blinding of patients was successful. The publicly available information about the study was changed after unblinding of the patients.

The physician was aware of the kind of briefing intervention he provided, but the acupuncturists were blinded to the briefing allocation of the patients and were not allowed to discuss the content of the briefing session with the patient. The statisticians were blinded to patient allocation when analyzing the data.

### Measures

For the assessment of the acupuncture treatment outcome expectation, the Expectation for Treatment Scale (ETS) was used.^31^ The total score ranges from 5 to 20, with higher values indicating higher expectation of a positive clinical outcome (Cronbach α, 0.77).

Pain intensity and pain bothersomeness were measured with an NRS^32^ from 0 to 10, with 0 indicating no pain or pain bothersomeness and 10 indicating worst pain or pain bothersomeness. The pain intensity and bothersomeness both refer to the last 7 days.

Adverse side effects were assessed using a 13-item self-report questionnaire with 3 levels of manifestation. The sum score after each acupuncture session could range from 0 to 39, with higher scores indicating more and/or stronger adverse side effects.

 Self-reported health was assessed with the 29-item short-form of the Patient-Reported Outcomes Measurement Information System (PROMIS)^33^ with 7 PROMIS domains (ie, depression, anxiety, physical function, pain interference, fatigue, sleep disturbance, and ability to participate in social roles and activities) and a single item on pain intensity.^34,35^ The total score of each domain was converted to a standardized T score.^33^

Optimism and pessimism were assessed using the Life Orientation Test–Revised (LOT-R)^36^ with 2 subscales (ie, optimism and pessimism). Each score can range from 0 to 12, with higher values indicating either higher optimism or pessimism (Cronbachα, optimism: 0.74; pessimism: 0.69).

To assess individuals’ sensitivity to medicines, we used the Perceived Sensitivity to Medicines scale (PSM).^37^ The total score ranges between 5 and 25, with higher scores pointing toward a higher perceived sensitivity to potential adverse side effects (Cronbach α, 0.91).

### Statistical Analysis

According to the statistical analysis plan (Supplement 1), we used a hierarchical test procedure in which the primary outcome (ETS score after the effectiveness briefing) was analyzed using analysis of covariance (ANCOVA), with the expectation briefing group as the variable of interest and ETS baseline and sex as covariates (eAppendix 6 in Supplement 2). In case of a statistically significant result, the second outcome (pain intensity after the minimal acupuncture treatment) was tested as confirmatory; otherwise, it was tested as exploratory using ANCOVA, with the expectation briefing as the variable of interest adjusted for pain intensity at the first visit, sex, treatment practitioner, and baseline optimism and pessimism. The adjusted means with 95% CIs are reported for each treatment group. Missing data (multiple imputations with 50 iterations according to Rubin^38^) were imputed with the predictive mean matching method in the case of continuous variables. For missing data in binary variables, a multinomial logit model was used for factors with more than 2 levels, and an ordered logit model was used for ordered factors (>2 levels).

For the analysis of adverse side effects, we compared the scores of each adverse side effect briefing group (regular vs intense) after session 1 to session 7. We expected overdispersion and zero-inflation and used a longitudinal zero-inflated negative binomial model with the adverse side effect briefing group, sex, and time from study entry as explanatory variables as well as patient-specific random intercepts (R package glmmTMB^39^). The results from the zero-inflated negative binomial regression were reported as the ratio of adverse side effect scores (ie, as the factor by which the adverse side effect score changes from the regular to the intense briefing) with 95% CIs and *P* values. A value of 1 thus corresponds to the null hypothesis that there is no effect of the briefing on the adverse side effect scores. All data analyses were performed using R statistical software^40^ version 3.6.1 with 2-sided tests. *P* < .05 was considered statistically significant.

## Results

Overall, 471 patients were assessed for eligibility, and 152 patients were randomized to 1 of 4 groups and analyzed (Figure). Of 152 patients (mean [SD] age, 39.54 [12.54] years), 100 (65.8%) were women, 86 (56.6%) attended school for at least 11 years, and 63 (41.4%) had a university degree. The 4 groups showed no relevant baseline differences (Table 1).

**Figure.**
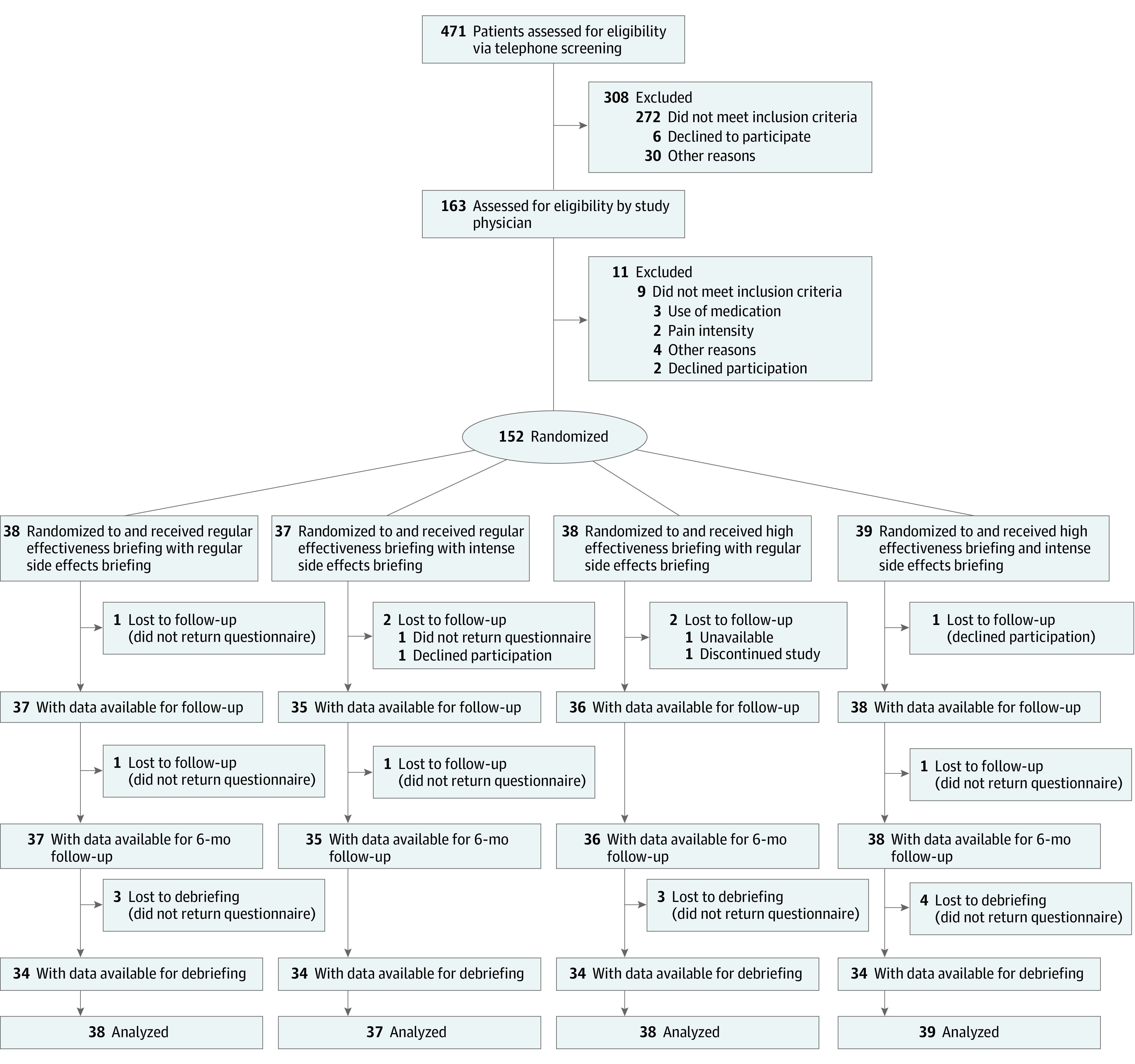
Study Flowchart

**Table 1.** Baseline Characteristics in the 4 Treatment Conditions

Characteristic	Patients, No. (%)			
	Regular expectation briefing		High expectation briefing	
	Regular adverse side effects briefing (n = 38)	Intense adverse side effects briefing (n = 37)	Regular adverse side effects briefing (n = 38)	Intense adverse side effects briefing (n = 39)
Sex				
Female	26 (68.4)	25 (67.6)	24 (63.2)	25 (64.1)
Male	12 (31.6)	12 (32.4)	14 (36.8)	14 (35.9)
Age, mean (SD), y	38.92 (13.60)	39.22 (10.31)	39.26 (13.46)	40.77 (12.80)
Education, No. (%)				
School ≤10 y	1 (2.6)	1 (2.7)	1 (2.6)	0 (0.0)
School ≥11 y	16 (42.1)	22 (59.5)	27 (71.1)	21 (53.8)
University	21 (55.3)	14 (37.8)	10 (26.3)	18 (46.2)
Employment status				
Employed	27 (71.1)	32 (86.5)	30 (78.9)	29 (74.4)
Unemployed	2 (5.3)	0 (0.0)	1 (2.6)	0 (0.0)
Student	7 (18.4)	2 (5.4)	5 (13.2)	9 (23.1)
Other	2 (5.3)	3 (8.1)	2 (5.3)	1 (2.6)
First language				
German	33 (86.8)	28 (75.7)	34 (89.5)	32 (82.1)
Other	5 (13.2)	9 (24.3)	4 (10.5)	7 (17.9)
Pain, mean (SD)				
Duration of pain, mo	77.50 (70.87)	98.00 (85.83)	87.63 (122.97)	122.90 (118.00)
Pain intensity, mean (SD)^a^	5.42 (1.45)	5.43 (1.46)	5.26 (1.16)	5.38 (1.29)
Pain bothersomeness, mean (SD)^a^	5.68 (1.92)	5.38 (2.13)	5.29 (1.78)	5.56 (1.92)
No additional pain medication	22 (57.9)	24 (64.9)	24 (63.2)	28 (71.8)
Additional pain medication	16 (42.1)	13 (35.1)	14 (36.8)	11 (28.2)
Previous experience				
Previous acupuncture treatment	14 (36.8)	21 (56.8)	15 (39.5)	19 (48.7)
No. of sessions of last acupuncture treatment, mean (SD)	6.43 (6.22)	7.90 (5.73)	6.50 (4.42)	6.41 (4.15)
Success of last acupuncture treatment, mean (SD)^b^	5.57 (3.08)	5.88 (3.36)	6.46 (2.99)	4.89 (3.08)
ETS, mean (SD)^c^	12.00 (3.20)	12.38 (3.38)	12.00 (3.54)	12.11 (2.96)
Self-reported PROMIS health T scores, mean (SD)				
Anxiety	53.57 (8.12)	54.95 (8.31)	54.07 (7.74)	55.71 (8.04)
Depression	52.08 (7.83)	51.82 (9.41)	51.77 (7.91)	53.44 (7.00)
Ability to participate in social roles	49.30 (7.18)	47.89 (7.11)	50.16 (7.82)	48.71 (8.16)
Fatigue	54.13 (8.78)	53.28 (8.93)	54.30 (8.63)	54.86 (10.30)
Pain interference	58.56 (5.10)	58.55 (5.21)	58.39 (4.31)	58.03 (4.48)
Physical functioning	46.02 (5.99)	45.08 (5.52)	46.48 (5.03)	47.95 (5.59)
Sleep disturbance	53.03 (9.50)	52.10 (9.72)	50.28 (6.74)	50.39 (8.76)
Personality score, mean (SD)				
Optimism^d^	8.71 (2.59)	8.51 (2.67)	8.70 (2.56)	9.03 (2.15)
Pessimism^d^	3.37 (2.34)	3.81 (2.09)	3.14 (2.21)	3.38 (2.55)
Perceived sensitivity to medicines^e^	10.74 (5.23)	10.00 (4.67)	11.11 (5.41)	12.13 (5.17)

Abbreviations: ETS, Expectation for Treatment Scale; PROMIS, Patient-Reported Outcomes Measurement Information System.

^a^ Measured on a numerical rating scale (range 0-10; higher scores indicate more pain or bothersomeness).

^b^ Measured on a numerical rating scale (range 0-10; higher scores indicating greater success).

^c^ ETS is scored from 5 to 20, with higher scores indicating greater expectations for success.

^d^ Measured with the Life Orientation Test–Revised (range, 0-12; higher scores indicate greater optimism or pessimism).

^e^ Measured with the Perceived Sensitivity to Medicine (range 5-25; higher scores indicate greater perceived sensitivity to medicine).

### Impact of the Briefing Interventions on Expectations and Pain

For the primary outcome of ETS score, patients in the regular expectation briefing group had an adjusted mean score of 12.58 (95% CI, 11.86 to 13.31) compared with 12.76 (95% CI 12.02 to 13.50) among patients in the high expectation briefing group. For pain intensity at the end of the treatment (NRS), patients in the regular expectation briefing group had an adjusted mean score of 4.49 (95% CI, 3.79 to 5.19) compared with 4.10 (95% CI, 3.38 to 4.82) among those in the high expectation briefing group. There was no evidence of any differences between the 2 expectation briefing groups for either ETS or pain intensity (Table 2). The findings for the expectation briefing did not substantially change when the adverse side effect briefing was included in the statistical model as a factor. At the 6-month follow-up, there was no evidence for differences in pain intensity between the regular and high expectation briefing groups (coefficient, −0.11; 95% CI, −0.72 to 0.51; *P* = .73).

**Table 2.** Expectation and Pain Scores for 2 Expectation Briefing Groups

Outcome	Adjusted mean (95% CI)		*P* value
	Regular expectation briefing (n = 75)	High expectation briefing (n = 77)	
Expectation, mean (95% CI)^a^	12.58 (11.86-13.31)	12.76 (12.02-13.50)	.60
Pain intensity (mean, 95% CI)^b^	4.50 (3.80-5.20)	4.12 (3.39-4.85)	.23

^a^ Expectation was measured using the Expectation for Treatment Scale (range, 5-20; higher scores indicate greater expectations for success).

^b^ Pain intensity was measured using a numeric rating scale (range, 0-10; higher scores indicate more intense pain).

### Impact of the Briefing Interventions on Adverse Side Effects

Patients with an intense adverse side effect briefing had a larger adverse side effects score by a factor of 1.31 (95% CI, 0.94-1.83) compared with patients with a regular adverse side effect briefing; however, this did not reach statistical significance (*P* = .11). When adjusting for sex, the estimated means in the 2 groups were not statistically different, although patients in the intense adverse side effects briefing group had a numerically higher number of reported adverse side effects (regular adverse side effects briefing group: 19.25; 95% CI, 13.41-27.64; intense adverse side effects briefing group: 25.43; 95% CI, 17.59-36.78; *P* = .07) (eAppendix 7 in Supplement 2). The result of the adverse side effect briefing did not differ between first time users of acupuncture and patients with previous acupuncture experience. No related serious adverse events were observed in any patient within the study.

### Blinding of Patients and Treatment Practitioners

The blinding of patients and treatment practitioner was successful (Table 3). However, most patients indicated in their posttreatment guess that they had received a regular expectation briefing.

**Table 3.** Patient and Treatment Practitioners Guesses Regarding Which Briefing a Patient Received vs Actual Allocation Groups

Perceived effectiveness briefing	Actual effectiveness briefing received by patients, No. (%)		Patients, total No.
	Regular	High	
Regular			
Patient	52 (38.5)	51 (37.8)	103
Treatment practitioner	47 (31.1)	51 (33.8)	98
High			
Patient	14 (10.4)	18 (13.3)	32
Treatment practitioner	27 (17.9)	26 (17.2)	53

## Discussion

This study, using a routine care scenario as a research model, did not confirm the hypothesis that the pretreatment briefing intervention about the effectiveness of an acupuncture treatment influences the treatment outcome expectations in patients with CLBP. There was no evidence that pain intensity after treatment was influenced by the pretreatment briefing on effectiveness. There was also no statistically significant evidence of an effect from the adverse side effect briefings; however, the study was not powered to confirm such differences between the adverse side effect briefing groups. For more conclusive findings, additional studies are required.

One could speculate that the missing impact of the effectiveness briefing on pain might be due to the low effectiveness of the acupuncture treatment. Compared with an earlier study with a similar setup, the magnitude of the pre-post treatment effects in our sample was very similar to the study from which the acupuncture intervention was derived.^25^ Brinkhaus et al^25^ found a pre-post effect size for pain intensity with an SMD of 0.96, which was comparable with the effect size (SMD, 0.91) in our study. Therefore, we can conclude that the acupuncture treatment was implemented successfully in our study, and the missing effect of the expectation briefing on pain intensity cannot be explained by missing treatment effects.

We were not able to confirm the findings of an earlier meta-analysis in pain research showing that positive messages improved treatment outcomes (SMD, −0.31).^9^ However, many trials included in this meta-analysis had nonblinded treatment practitioners, and some were not fully randomized. After the exclusion of potentially biased trials, there was no longer any evidence of an effect.

Two trials are closely related to our investigation. An earlier study using sham acupuncture^23^ found an effect of communication on treatment outcomes. However, the communication in the control group was described as limited, which raises the question of whether this kind of communication reflects regular practice and is credible to inform routine care. A study by Suarez et al^41^ also used positive suggestions during acupuncture treatment and found beneficial effects of positive verbal suggestions. There was a statistically significant difference between acupuncture and sham acupuncture, but the communication style had a small effect (SMD, 0.20) on the treatment effects in both groups. This study had some similarities to our study, but it was less conceptional and standardized, and it is unclear whether the effects could be attributed to restricted communication or empathic communication. Our study specifically aimed to use a treatment and briefing model that was similar to routine practice, and we used a standardized intervention to manipulate communication before the treatment to disentangle pretreatment and within-treatment communication.

In our study, the magnitude of the nocebo effect of the intervention was stronger than that of the placebo effect. This is in line with other pain studies in which the magnitude of nocebo effects after an alteration of expectation was large (SMD, 0.81).^42^ However, the magnitude of nocebo effects is variable and depends on contextual factors of the study (eg, setting, participants).^43^ In experimental research, it has been well documented that suggestions of symptoms, a high sensitivity to medication,^44^ and personality traits (ie, anxiety)^45^ can increase the likelihood of nocebo responses. These findings indicate that informing patients about the adverse side effects of a treatment might need more attention.^46^ Experts have suggested that positive framing of adverse side effects might be a suitable option within the given ethical framework.^47^

In placebo research, there is still a huge gap between the number of experimental studies using suggestions and the number of studies in a real-world clinical setting using pretreatment briefing information. Our findings imply that physicians’ communication about treatment effectiveness before treatment may not have as much impact on treatment outcome expectations as is generally assumed. This might be regarded by physicians as a relief given that they are already burdened with many challenges (ie, multimorbidity, interaction effects of drugs) when seeing patients in very limited time. However, more studies in a real-world context are necessary to provide more robust evidence about briefings for treatment outcomes because the findings are still heterogeneous. Considering that our study suggested a stronger influence of pretreatment information on the nocebo response, this field should receive more attention in upcoming studies because communication about adverse side effects is a regular task for physicians.

### Strengths and Limitations

We would like to emphasize some important features of this study. The blinding of our treatment practitioners is a strength because our study was able to differentiate between pretreatment information (ie, briefing) and within-treatment communication. The treatment practitioners, patients, and statisticians were blinded. The assessment of expectations after the briefing is an important strength given that similar studies claimed that the change in expectations act as a mechanism for the change in clinical outcomes, but they had not assessed expectations.^41^

This study also has limitations. The generalizability of our study might be affected by the type of study intervention, the recruitment strategy, and the rather high education level of the patients in our sample. We conducted several sensitivity analyses to test the robustness of our findings (eg, impact of prior acupuncture or treatment adherence on outcomes), but none of the additional variables changed the overall findings, which could also be caused by the low sample size in subgroups.

## Conclusions

These findings indicate that briefings about treatment benefits (ie, placebo) when referring to a treatment in routine care might not be as important as previously thought. However, information about adverse side effects might require more research to understand nocebo responses.

## Notes

Supplement 1.
Trial Protocol and Statistical Analysis Plan

Supplement 2. **eAppendix 1.** Sample Size Calculation **eAppendix 2.** Exclusion Criteria **eAppendix 3. **Content of Different Effectiveness Briefings Before Minimal Acupuncture Treatment, in the Booster Session (Verbal), and in Booster Emails **eAppendix 4.** Description of Acupuncture Treatment **eAppendix 5.** Acupuncture Regimen **eAppendix 6.** Data Analysis **eAppendix 7.** Type of Adverse Side Effects per Session, Stratified by Briefing

Supplement 3.
Data Sharing Statement
