# Supplementary material for: Effect of Briefing on Acupuncture Treatment Outcome Expectations, Pain, and Adverse Side Effects Among Patients With Chronic Low Back Pain: A Randomized Clinical Trial
Source: JAMA Netw Open. 2021 Sep 10;4(9):e2121418. doi: 10.1001/jamanetworkopen.2021.21418 (PMC8433606; doi:10.1001/jamanetworkopen.2021.21418)
Supplement: Supplement 1. — Trial Protocol and Statistical Analysis Plan [file jamanetwopen-e2121418-s001.pdf]

University of Zurich[UZH]

Epidemiology, Biostatistics
and Prevention Institute,
Biostatistics Department
+41 44 634 46 41
www.biostat.uzh.ch

STATISTICAL ANALYSIS PLAN

Changing Patients' Expectations to Increase Treatment Effects: A Randomized Controlled Study in Chronic Low Back Pain Patients Receiving Acupuncture

Short Title: Pain Acupuncture Treatment Expectation (PATE)

Principal Investigators: Claudia M. Witt (PI), Jürgen Barth (Co-PI)

Statisticians:
Leonhard Held (*leonhard.held@uzh.ch*)
Niels Hagenbuch (*niels.hagenbuch@uzh.ch*)
Stefanie Muff (*stefanie.muff@uzh.ch*)

Version 1.1 of June 20, 2018

DISCLAIMER

This report was written by the statistical consulting service of the University of Zurich, Epidemiology, Biostatistics and Prevention Institute, Biostatistics Department (IEBP) in collaboration with the Institute for Complementary and Integrative Medicine (IKI). The authors agree that they follow the ICMJE guidelines for authorship, http://www.icmje.org.

Contents

0 Preface 5

1 Administrative Information 5
- 1.1 Title and Registration 5
- 1.2 SAP Version 5
- 1.3 Protocol Version 5
- 1.4 SAP Revision History 5
- 1.5 Roles and Responsibilities 5
- 1.6 Signatures and Dates 6

2 Introduction 6
- 2.1 Background and Rationale 6
- 2.2 Objectives 7

3 Study Methods 7
3.1 Trial Design 7
3.2 Randomization 7
3.3 Sample Size 7
3.3.1 Research Question 1a 7
3.3.2 Research Question 1b 7
3.3.3 Research Question 2 8
3.4 Framework and Hypotheses 8
3.4.1 Research Question 1a 8
3.4.2 Research Question 1b 8
3.4.3 Research Question 2 8
3.5 Statistical Interim Analyses and Stopping Guidance 8
3.6 Timing of Final Analysis 8
3.7 Timing of Outcome Assessment 9

4 Statistical Principles 9
4.1 Confidence Intervals and p-Values 9
4.2 Adherence and Protocol Deviations 9
4.3 Analysis Population 9

5 Trial Population 9
5.1 Screening Data 9
5.2 Eligibility 9
5.3 Recruitment 10
5.4 Withdrawal/Follow-up 10
5.5 Baseline Patient Characteristics 10

6 Analysis 10
6.1 Outcome Definitions 10
6.2 Analysis Methods 10
6.2.1 Research Question 1a: The Expectation of Acupuncture Treatment (EAT) 10
6.2.2 Research Question 1b: Average Pain Intensity at the End of Treatment 11
6.2.3 Research Question 2: Side Effects Score at the End of Treatment 11
6.3 Assumptions 12
6.4 Sensitivity Analyses 12
6.4.1 Per-Protocol Analyses: Research Questions 1a and 1b 12
6.4.2 Protocol-Deviations: Research Question 1b 12
6.4.3 Extended Models: Research Questions 1a, 1b, and 2 12
6.5 Missing Data 12
6.6 Analyses of Secondary Outcomes 14
6.6.1 Research question 1a 14
6.6.2 Research question 1b 14
6.6.3 Research question 2 14
6.6.4 2 x 2 Factorial Analyses 15
6.6.5 Pain Intensity, Follow-Up 15
6.6.6 Perceived Sensitivity to Medicines Score, PSM 16
6.6.7 Post-treatment Guess 16
6.7 Further Analyses 16

6.8 Harms 16
6.9 Statistical Software 16

7 Separate Analyses **16**

8 References **17**

A Additional Analyses of Secondary Outcomes **18**
A.1 Baseline With Covariates 18
A.2 Baseline With Additional Covariates 18
A.3 Follow-Up With Covariates 19
A.4 Follow-Up With Additional Covariates 20

B Glossary of Variable Names **21**

Abbreviations

ANCOVA	Analysis of covariance
CTC	Clinical trial center, University Hospital Zurich (USZ)
DRKS	Deutsches Register klinischer Studien
EAT	Expectation of acupuncture treatment
FPFV	First patient first visit
INLA	Integrated nested Laplace approximations
IQR	Interquartile range
LPLV	Last patient last visit
MAR	Missing at random
MCAR	Missing completely at random
MCMC	Markov Chain Monte Carlo
MICE	Multivariate imputation by chained equations
NA	Not applicable
NRS	Numeric rating scale
OS	Operating system
PMM	Predictive mean matching
SAP	Statistical analysis plan
SD	Standard deviation

0 Preface

This statistical analysis plan has been written following Gamble et al. (2017).

1 Administrative Information

1.1 Title and Registration

Statistical analysis plan (SAP) for the trial *Changing Patients' Expectations to Increase Treatment Effects: A Randomized Controlled Study in Chronic Low Back Pain Patients Receiving Acupuncture. Short Title: Pain Acupuncture Treatment Expectation (PATE).*

Trial registration: DRKS00010191 (Deutsches Register klinischer Studien, `www.drks.de`), registration date 30 March 2016.

1.2 SAP Version

Version 1.0, May 29, 2018.

1.3 Protocol Version

This document is based on Study Protocol Version 2, dated 9 February 2016.

1.4 SAP Revision History

Prev. Version	Updated Version	Sections Changed	Description and Reason for Change	Date of Change
	0.0		Creation of document	14.02.2018
0.0	1.0		First final version	29.05.2018

1.5 Roles and Responsibilities

- Prof. Dr. Leonhard Held, Head of Dept. of Biostatistics at the Epidemiology, Biostatistics and Prevention Institute, University of Zurich

  Role: Senior statistician overseeing the design and analysis of the trial

- Dr. med., MSc Statistics ETH Niels Hagenbuch, Scientific Staff at the Epidemiology, Biostatistics and Prevention Institute, University of Zurich

  Role: Writing of the SAP (up to version 1.0) and developing preliminary R code.

- Dr. sc. nat. Stefanie Muff, Scientific Staff at the Epidemiology, Biostatistics and Prevention Institute, University of Zurich

  Role: Analysis of the data.

- Prof. Dr. med. Claudia M. Witt, MBA, Institute for Complementary and Integrative Medicine, University Hospital Zurich (USZ)

  Role: Principal investigator

- PD Dr. phil. Jürgen Barth, Institute for Complementary and Integrative Medicine, University Hospital Zurich (USZ)

  Role: Principal co-investigator

- Anja Zieger, B. A., Institute for Complementary and Integrative Medicine, University Hospital Zurich (USZ)

  Role: PhD Student, analysis of data (described in a separate SAP)

- Alexandra Kern, MSc, Institute for Complementary and Integrative Medicine, University Hospital Zurich (USZ)

  Role: PhD Student, analysis of data (described in a separate SAP)

- Dr. rer. nat. Stefanie Maier, Institute for Complementary and Integrative Medicine, University Hospital Zurich (USZ)

  Role: Study Coordinator

1.6 Signatures and Dates

[illegible] 25.6.2018

Prof. Dr. Leonhard Held (Senior Statistician)

[illegible] 27.6.2018

Dr. Niels Hagenbuch (Scientific Staff)

S. Muff 2.7.2018

Dr. Stefanie Muff (Scientific Staff)

[illegible] 21.6.18

Prof. Dr. med. Claudia M. Witt (Principal Investigator)

[illegible] 21.06.2018

PD Dr. phil. Jürgen Barth (Principal Co-Investigator)

[illegible] 21.06.2018

Anja Zieger, B. A. (Scientific Staff)

A Kern 22.6.2018

Alexandra Kern, MSc (Scientific Staff)

[illegible] 21.6.18

Dr. rer. nat. Stefanie Maier (Study Coordinator)

2 Introduction

2.1 Background and Rationale

See Study Protocol, Section 3, Introduction, pp. 19–22.

2.2 Objectives

This study wants to manipulate pre-treatment expectations in patients receiving acupuncture, and to determine whether expectation can mediate treatment outcomes. The impact of two verbal briefing interventions (one on effectiveness and one on side effects) upon patients' treatment expectation before acupuncture treatment, the experience of side effects during treatment, and pain reduction after treatment will be analysed.

3 Study Methods

3.1 Trial Design

The trial is a randomized, double-blind (patients and statisticians), four-armed trial with a 2 x 2 factorial design. The factors are effectiveness briefing (regular *vs.* high) and side effects briefing (regular *vs.* intense). The statistical analysis will be conducted blind for allocation of patients.

3.2 Randomization

Block-randomization with randomly varying block sizes (either 4 or 8) in a 1:1:1:1 ratio was used. An additional block will be used at the beginning that is not balanced, this means that the final totals in each group may not be exactly equal (but still similar). This made it more difficult to anticipate future assignments as the numbers will not return to equality at the end of each block (Schulz and Grimes, 2002). The randomization sequence was stratified by gender using R (version 3.1.0). For allocation of patients via the internet, the software secuTrial® (Clinical Trial Center, University Hospital Zurich) was used.

3.3 Sample Size

For sample size calculation, nQuery Advisor (version 7.0) was used.

3.3.1 Research Question 1a

No previous data on expectancy change were available for trial planning purposes. For this study, the assumption was that the high expectation briefing is clinically meaningful better than a regular expectancy briefing in changing expectation, with a standardized mean difference (SMD) of 0.5. Assuming a power of 80% and a two-sided alpha level of 5%, a sample size of 128 patients (64 high expectation briefing and 64 regular expectation briefing) would enable the detection of a difference of SMD 0.5 for the expectancy briefing intervention. Assuming a dropout rate of approximately 15%, 150 patients were sought to be enrolled.

3.3.2 Research Question 1b

With the above mentioned sample size the power is 56–65% ($n = 128–150$) to detect a significant difference between high and regular expectation briefing on pain intensity after the acupuncture treatment. This assumption is based on a previous study (Linde et al., 2007) with response rates of 54% in the high expectation group and 39% in the low expectation group that resulted in a SMD of 0.38.

3.3.3 Research Question 2

Variation of the outcomes was not known prior to the study. Thus, the sample size was determined using two-group Fisher's exact tests of equal proportions (for binary outcomes). 50% of the patients in the intense safety briefing group and 10% in the regular group were expected to have side effects such that a side effects score can be calculated (Witt et al., 2009). It showed that with 64 patients in each study group such differences in outcome scores could be detected with at least 99% power at a 0.05 two-sided significance level. This power analysis was conservative and the actual power of the study will be higher since a count score and not a binary variable is used.

3.4 Framework and Hypotheses

In a first step, research question 1a will be tested. Only if the high expectation briefing significantly changes the expectations (rejection of null hypothesis 1a), research question 1b concerning the effect on pain intensity will be tested. In both tests, the same significance level of $\alpha = 0.05$ will be used (see also Section 4.1). This hierarchical order ensures to control for multiple testing.

3.4.1 Research Question 1a

Null hypothesis:

Expectation after high efficacy briefing = regular efficacy briefing.

The alternative hypothesis is:

Expectation after high efficacy briefing ≠ regular efficacy briefing.

3.4.2 Research Question 1b

Null hypothesis:

Pain intensity at the end of acupuncture treatment after high efficacy briefing = pain intensity after regular briefing.

The alternative hypothesis is:

Pain intensity at the end of acupuncture treatment after high efficacy briefing ≠ pain intensity after regular briefing.

3.4.3 Research Question 2

Null hypothesis:

Side effects after intense side effect briefing = side effects after regular side effect briefing.

The alternative hypothesis is:

Side effects after intense side effect briefing ≠ side effects after regular side effect briefing.

3.5 Statistical Interim Analyses and Stopping Guidance

There will be no interim analyses.

3.6 Timing of Final Analysis

After the information was revealed that the actual treatment in the study were different ways of briefing and not the acupuncture (cf. Study Protocol, Section 3.6, Risk/Benefits, p. 21f., and Section 6.2.2, Blinding Procedures, p. 25), the patients could opt to have their data deleted. Data from each

patient is included in the analysis if there is no withdrawal within 8 weeks after unblinding (which took place on 23 January 2018).

3.7 Timing of Outcome Assessment

The last patient visit was on 11 December 2017. The mailing of the follow-up questionnaires will be on 7 May 2018. For further details, see Study Protocol, Section 9, Study Procedures, pp. 30ff.

4 Statistical Principles

4.1 Confidence Intervals and p-Values

All confidence intervals are 95% and two-sided. All tests are two-sided and performed at a significance level of 0.05.

4.2 Adherence and Protocol Deviations

A few patients did not fill in the baseline questionnaire. Baseline values are, therefore, missing. A few patients stopped the acupuncture treatment prematurely and did not receive the foreseen eight acupuncture session. All patients received the briefing intervention as foreseen. Some patients used opioids between acupuncture sessions, which was initially an exclusion criteria at baseline if used on a regular basis.

4.3 Analysis Population

- For research question 1a, there are very few patients who did not fill in the baseline questionnaire An intention-to-treat analysis will be performed using multiple imputation (cf. Section 6.5) to handle missing values.
- For research questions 1b, an intention-to-treat analysis will be performed using multiple imputation (cf. Section 6.5) to handle missing values.
- For research questions 2, an intention-to-treat analysis will be performed (no missing data handling needed, cf. Section 6.2.3).
- In a sensitivity analysis (cf. Section 6.4.1), per-protocol analyses will be done using a complete-case data set (research questions 1a and 1b). In addition, to assess the influence of protocol deviations (cf. 6.4.2), the analyses will be repeated without patients that used opioides (for research question 1b), and only with patients that had at least 6 out of the 8 acupuncture treatment sessions (for research question 1b), respectively.

5 Trial Population

5.1 Screening Data

See Study Protocol, Section 7.2, Recruitment and Screening, p. 26.

5.2 Eligibility

See Study Protocol, Section 7.1, Eligibility Criteria, pp. 25f.

5.3 Recruitment

See Study Protocol, Section 7.2, Recruitment and Screening, p. 26.

5.4 Withdrawal/Follow-up

See Study Protocol, Section 7.4, Criteria for Withdrawal/Discontinuation of Participants, p. 26.

5.5 Baseline Patient Characteristics

All variables used in the statistical analysis will be summarized. Continuous data will be summarized by mean, standard deviation (SD), minimum, maximum, 1st quartile, median, 3rd quartile, interquartile range, number of observations used, and number of observations missing. Categorical data will be summarized by numbers and percentages. This description will be done for the total sample and the four randomized groups respectively.

6 Analysis

6.1 Outcome Definitions

The three primary outcomes are

1a) Expectation of acupuncture treatment (questionnaire "P2 EAT", now called ETS, but will be labelled with EAT in this SAP), a score based on 5 questions having each 4 levels. It can range from 5 to 20. The questionnaire is filled in by the patients at baseline before the treatment (*i. e.* the briefing by the study doctor), immediately after the treatment, and once more after the 4th acupuncture session (in week 2).

1b) Average pain intensity over the last 7 days after 4 weeks at the end of the treatment (questionnaire "P7.2 NRS"). As a measure, a numeric rating scale (NRS) is used in a self-reporting fashion. The NRS has 11 discrete levels from 0 (no pain) to 10 (worst pain possible). The NRS is filled in at baseline (before the treatment), after two weeks, and at the end of the acupuncture sessions. Note that due to eligibility criteria, only patients with an NRS value ≥ 4 were included in the study. Accordingly, the baseline values of pain intensity lie within the range of 4–10.

2) The side effects score after each acupuncture session (questionnaire "P9 Nebenwirkungen"). A self-report questionnaire with 13 questions and 4 levels each (0 to 3) is used, thus the values range from 0 to 39 per session. As a secondary outcome, the total side effect score for each patient, adjusted for the number of acupuncture sessions that the patient actually received, will be used.

6.2 Analysis Methods

6.2.1 Research Question 1a: The Expectation of Acupuncture Treatment (EAT)

The EAT score will be analyzed using analysis of covariance (ANCOVA). Outcome variable is the EAT score (`EAT_1b`) obtained immediately after the effectiveness briefing (regular or high). Covariate of interest is the effectiveness briefing group (`EXPBRIEF`). As adjustment variables, EAT at baseline (`EAT_BL`), and gender (`pers_1_2`) will be used.

Missing values will be imputed (see Section 6.5). As a sensitivity analysis, the same model will be fitted again, using only complete cases (see Section 6.4). The estimated mean difference of the two briefing groups with 95% confidence interval and p-value will be reported. The estimated effects of the adjusting variables will not be shown. In addition, adjusted means with 95% confidence intervals will be reported for each treatment group, where EAT at baseline will be fixed at its mean, and the two genders effects will be weighted with 0.5.

6.2.2 Research Question 1b: Average Pain Intensity at the End of Treatment

If the result of the analysis of research question 1a indicates a significant effect of briefing on the expectations of acupuncture treatment, research question 1b will be analysed. The pain intensity will be analyzed using ANCOVA. Outcome variable is the pain intensity (`nrs_8_1`) measured at the end of the acupuncture treatment. Covariate of interest is the effectiveness briefing group (`EXPBRIEF`). As adjustment variables, pain intensity at first visit (`screening_1a_4a`), gender (`pers_1_2`), acupuncture therapist (`acupuncturist`), and baseline optimism (`LOT_BL_optim`) and pessimism (`LOT_BL_pessim`) will be used.

Missing values will be imputed (see Section 6.5). As a sensitivity analysis, the same model will be fitted again, using only complete cases (see Section 6.4). The estimated mean difference of the two briefing groups with 95% confidence interval and p-value will be reported. The estimated effects of the adjusting variables will not be shown. In addition, adjusted means with 95% confidence intervals will be reported for each treatment group, where all continuous adjustment variables will be fixed at their mean, and the effects of acupuncture therapist and gender will be given equal weights over the factor levels (thus 0.5 for gender and 0.33 for acupuncturist).

6.2.3 Research Question 2: Side Effects Score at the End of Treatment

As a primary analysis, the side effect scores that are available from up to 7 measurements (before each session, except session 1) will be compared between side effects briefing groups. Covariate of interest thus is the side effects briefing group (`SIDEBRIEF`). The side effect scores will be treated as counts, because they are expected to show a skewed distribution. Because each patient has up to 7 outcome values, a longitudinal zero-inflated negative binomial model will be used. This model will provide increased statistical power with respect to a model with total score as outcome, because it utilizes all outcomes of all patients.

In addition to the briefing group, the linear predictor of the model includes gender (`pers_1_2`) and the time of the measurement since the first session (in days, derived from session dates `verlaufdokua_1b`, `verlaufdokua_2`, ..., `verlaufdokua_8`) as additional fixed effects, and patient-specific random effects for the intercept and time, where the latter accounts for patient-specific dependencies (*i.e.* slopes) on time. The zero-inflation probability will modelled as dependent on `SIDEBRIEF`. The model will be fitted with the `glmmTMB()` function from the R package `glmmTMB` (Brooks et al., 2017). If this longitudinal mixed model fails to converge with the specification given here, the random effect for time will be removed, that is, the dependency on time is then assumed to be the same for all patients.

It will be assumed that missing side effect scores are missing at random (MAR), that is, missingness depends only on observed variables. Importantly, the longitudinal analysis allows for unbalanced data, thus MAR in the outcome is automatically properly accounted for and does thus not require any imputation (Ibrahim and Molenberghs, 2009).

The result from the zero-inflated negative binomial regression will be reported as the ratio of side effect scores (*i.e.*, as the *factor* by which the side effect score changes from regular to intense

side effects briefing) with 95% confidence interval and p-value. Note that, in this case, a ratio of 1 corresponds to the null hypothesis that there is no effect of `SIDEBRIEF` on the side effect scores. The estimated effects of the adjusting variables will not be shown.

6.3 Assumptions

The EAT score (baseline and follow-up) and the pain intensity at first visit and follow-up are treated as continuous.

All relevant variables are analyzed graphically by plotting the histograms and boxplots, split by the 2 x 2 groups. The results will be presented in a separate document.

6.4 Sensitivity Analyses

6.4.1 Per-Protocol Analyses: Research Questions 1a and 1b

Research questions 1a and 1b will be re-analysed per-protocol as well. Missing values will not be imputed, incomplete cases will be dropped. The models will be the same as described in Sections 6.2.1 and 6.2.2.

6.4.2 Protocol-Deviations: Research Question 1b

Research question 1b will be repeated excluding the patients who used opioids. The analysis of research question 1b will also be repeated including only patients that had at least 6 of the 8 acupuncture treatment sessions.

6.4.3 Extended Models: Research Questions 1a, 1b, and 2

All research questions (1a, 1b, and 2) will also be analyzed using an extended model including age (`age`), duration of pain (`painduration`), depression (`PROMIS_BL_depression.theta`), and CARE score (`CARE_1a_red1`) as additional covariates. Missing values will be imputed.

The follow-up pain intensity (see Section 6.6.5) will be re-analyzed with an extended model as well, including age (`age`), duration of pain (`painduration`), depression (`PROMIS_BL_depression.theta`), and CARE score (`CARE_1a_red1`) as additional covariates. Missing values will be imputed.

Confidence intervals and p-values from sensitivity analyses are considered explorative.

6.5 Missing Data

The analysis will require to impute missing values, intermittent missing side effects scores, and dropouts.

The distribution and pattern of missing values will be assessed using tables and plots (using the R package **VIM**; Templ et al. (2017)), giving first hints whether the missing completely at random (MCAR) assumption holds.

Type of missingness Missing at random (MAR) is assumed, and data will be imputed applying multivariate imputation by chained equations (MICE) using the R package **mice** (van Buuren and Groothuis-Oudshoorn (2011); van Buuren (2012)).

Imputation model Imputations for continuous data will be generated with the predictive mean matching method (pmm). For binary data a logistic regression model, for factors with more than two levels a multinomial logit model, and for ordered factors (>2 levels), an ordered logit model will be used.

Predictor variables For the analysis of **Research Question 1a** and the concomitant sensitivity analyses, the following variables will be used as predictors in the imputation process of missing values:

- EAT score obtained immediately after the effectiveness briefing (`EAT_1b`)
- effectiveness briefing group (`EXPBRIEF`)
- side effects briefing group (`SIDEBRIEF`)
- EAT at baseline (`EAT_BL`)
- gender (`pers_1_2`)
- age (`age`)
- duration of pain at first visit (`painduration`)
- pain bothersomeness at baseline (`nrs_1a_2`)
- depression (`PROMIS_BL_depression.theta`)
- CARE score (`CARE_1a_red1`)

For the analysis of **Research Question 1b** and the concomitant sensitivity analyses, the following variables will be used as predictors in the imputation process of missing values:

- pain intensity measured at the end of the acupuncture treatment (`nrs_8_1`)
- effectiveness briefing group (`EXPBRIEF`)
- side effects briefing group (`SIDEBRIEF`)
- pain intensity at first visit (`screeningb_1a_4a`)
- baseline optimism (`LOT_BL_optim`)
- baseline pessimism (`LOT_BL_pessim`)
- acupuncture therapist (`acupuncturist`)
- gender (`pers_1_2`)
- age (`age`)
- duration of pain at first visit (`painduration`)
- pain bothersomeness at baseline (`nrs_1a_2`)
- depression (`PROMIS_BL_depression.theta`)
- CARE score (`CARE_1a_red1`)

Variables that are functions of other (incomplete) variables There is no transformation, combination, or recoding of any variable involved. Hence, no passive imputation or post-processing of imputed values is required.

Order in which variables will be imputed Since there are no interactions or transformations, the visiting scheme is not important (except for efficiency) as long as each variable in the data set is visited often enough. Setting the number of iterations to 50 should preclude any issues with respect to visiting (see van Buuren and Groothuis-Oudshoorn (2011), Section 3.6).

Starting imputations and number of iterations The number of iterations will be set to 50. To facilitate reproducibility, a seed for the random number generator will be set for all steps involving a random mechanism.

Number of imputed data sets The number of imputation will be set to $m = 50$ in order to minimize simulation error.

Analysis model The analysis models are described in Section 6.2.

Reported values Pooled results, *i.e.* the average of the estimated coefficients (slope or effect), for multiple imputation adjusted confidence intervals (Rubin, 2004, pp. 76–77), p-values, the fraction of missing information, and the proportion of total variance attributable to missing data will be reported.

6.6 Analyses of Secondary Outcomes

6.6.1 Research question 1a

A second ANCOVA using the EAT score obtained after the 4th acupuncture session (in week 2) as outcome will be performed. The covariates and adjusting variables will be as described in Section 6.2.1.

6.6.2 Research question 1b

A second ANCOVA using the pain intensity obtained after the 4th acupuncture session (in week 2) as outcome will be performed. The covariates and adjusting variables will be as described in Section 6.2.2.

6.6.3 Research question 2

A secondary outcome for research question 2 is the total side effect score for each patient, which will be adjusted for the number of acupuncture sessions that the patient actually received minus 1. For a patient with the number of sessions given by N_s, the outcome is thus

$$\text{Total score} = \frac{(\text{Score}_1 + \text{Score}_2 \ldots + \text{Score}_{N_s - 1})}{N_s - 1} \cdot 7 \,.$$

As this may lead to total score values that are non-integers, the total score will be rounded to the next integer. A zero-inflated negative binomial model with `SIDEBRIEF` and gender (`pers_1_2`) will be

used. As for the primary analysis (Section 6.2.3), the result from the zero-inflated negative binomial regression will be reported as the ratio of side effect scores with 95% confidence interval and p-value. In addition, adjusted means of the total side-effects scores with 95% confidence intervals will be reported for each treatment group, where the gender effect will be weighted with 0.5.

6.6.4 2 x 2 Factorial Analyses

Two additional analyses of research questions 1a and 1b will be performed using the two main factors effectiveness briefing (`EXPBRIEF`) and side effects briefing (`SIDEBRIEF`) together in the same model. Missing values will be imputed.

6.6.5 Pain Intensity, Follow-Up

For research question 1b, the follow-up measurement of pain (outcome variable `nrs_fu_1`) will be analysed as well, using the same method as described in Section 6.2.2. For the imputation of missing values, the following predictor variables will be used:

- pain intensity measured at follow-up (`nrs_fu_1`)
- effectiveness briefing group (`EXPBRIEF`)
- side effects briefing group (`SIDEBRIEF`)
- pain intensity at first visit (`screeningb_1a_4a`)
- pain intensity after acupuncture treatment (`nrs_8_1`)
- baseline optimism (`LOT_BL_optim`)
- baseline pessimism (`LOT_BL_pessim`)
- acupuncture therapist (`acupuncturist`)
- gender (`pers_1_2`)
- age (`age`)
- duration of pain at first visit (`painduration`)
- pain bothersomeness at baseline (`nrs_1a_2`)
- pain bothersomeness at the end of acupuncture treatment (`nrs_8_2`)
- depression (`PROMIS_BL_depression.theta`)
- CARE score (`CARE_1a_red1`)

6.6.6 Perceived Sensitivity to Medicines Score, PSM

The PSM score will be analyzed using three ANCOVA models (no imputation):

```
PSM_8 ~ SIDEBRIEF + pers_1_2 + PSM_BL

PSM_8 ~ SIDEBRIEF + pers_1_2 + LOT_BL_optim + LOT_BL_pessim +
acupuncturist + PSM_BL

PSM_8 ~ SIDEBRIEF + pers_1_2 + LOT_BL_optim + LOT_BL_pessim +
acupuncturist + age + painduration + CARE_1a_red1 + PSM_BL
```

6.6.7 Post-treatment Guess

After the study, patients and acupuncturists were asked to guess in which effectiveness briefing and in which side effect briefing group they had been randomized.

The agreement between the patients and the acupuncturists will be assessed using four chi-square (χ^2) tests:

- `postguessp_8_1` *vs.* `EXPBRIEF`
- `postguessa_8_1` *vs.* `EXPBRIEF`
- `postguessp_8_3` *vs.* `SIDEBRIEF`
- `postguessa_8_3` *vs.* `SIDEBRIEF`

6.7 Further Analyses

A list of further inquiries into the data set, defined by Jürgen Barth and Anja Zieger, is provided in Appendix A. The models to be fitted are described in R syntax. A glossary of the variable names is provided in Appendix B.

6.8 Harms

The side effects of the acupuncture treatment are central to research question 2 and will be analyzed as discussed in Section 6.2.

6.9 Statistical Software

The analyses described in this SAP will be performed in the R programming language (R Core Team, 2017) (version 3.4.4) running on Linux Ubuntu 16.04 LTS.

Required R packages are mentioned in the respective sections of this document.

7 Separate Analyses

Additional analyses will be conducted within the PhD project by Anja Zieger and Alexandra Kern. Separate SAPs will be written.

8 References

Brooks, M. E., Kristensen, K., van Benthem, K. J., Magnusson, A., Berg, C. W., Nielsen, A., Skaug, H. J., Mächler, M. and Bolker, B. M. (2017). glmmTMB balances speed and flexibility among packages for zero-inflated generalized linear mixed modeling. *The R Journal* **9** 378 – 400.

Gamble, C., Krishan, A., Stocken, D., Lewis, S., Juszczak, E., Doré, C., Williamson, P. R., Altman, D. G., Montgomery, A., Lim, P., Berlin, J., Senn, S., Day, S., Barbachano, Y. and Loder, E. (2017). Guidelines for the content of statistical analysis plans in clinical trials. *JAMA* **318** 2337–2343.

Ibrahim, J. G. and Molenberghs, G. (2009). Missing data methods in longitudinal studies: a review. *Test (Madr.)* **18** 1–45.

Linde, K., Witt, C. M., Streng, A., Weidenhammer, W., Wagenpfeil, S., Brinkhaus, B., Willich, S. N. and Melchart, D. (2007). The impact of patient expectations on outcomes in four randomized controlled trials of acupuncture in patients with chronic pain. *Pain* **128** 264–271.

R Core Team (2017). *R: A Language and Environment for Statistical Computing*. R Foundation for Statistical Computing, Vienna, Austria.
URL `https://www.R-project.org/`

Rubin, D. B. (2004). *Multiple Imputation for Nonresponse in Surveys*. Wiley classics library edition ed. Wiley Classics Library, Wiley.

Schulz, K. F. and Grimes, D. A. (2002). Unequal group sizes in randomised trials: guarding against guessing. *The Lancet* **359** 966–970.

Templ, M., Alfons, A., Kowarik, A. and Prantner, B. (2017). VIM: Visualization and imputation of missing values. R package version 4.7.0.
URL `https://cran.r-project.org/web/packages/VIM/index.html`

van Buuren, S. (2012). *Flexible Imputation of Missing Data*. 1st ed. CRC Press.

van Buuren, S. and Groothuis-Oudshoorn, K. (2011). mice: Multivariate imputation by chained equations in r. *Journal of Statistical Software* **45** 1–67.
URL `https://www.jstatsoft.org/article/view/v045i03`

Witt, C. M., Pach, D., Brinkhaus, B., Wruck, K., Tag, B., Mank, S. and Willich, S. N. (2009). Safety of acupuncture: results of a prospective observational study with 229,230 patients and introduction of a medical information and consent form. *Forschende Komplementärmedizin* **16** 91–97.

A Additional Analyses of Secondary Outcomes

A.1 Baseline With Covariates

```
PROMIS_8_anxiety.theta ~ EXPBRIEF + pers_1_2 + LOT_BL_optim +
LOT_BL_pessim + acupuncturist + PROMIS_BL_anxiety.theta

PROMIS_8_depression.theta ~ EXPBRIEF + pers_1_2 + LOT_BL_optim +
LOT_BL_pessim + acupuncturist + PROMIS_BL_depression.theta

PROMIS_8_fatigue.theta ~ EXPBRIEF + pers_1_2 + LOT_BL_optim +
LOT_BL_pessim + acupuncturist + PROMIS_BL_fatigue.theta

PROMIS_8_paininterference.theta ~ EXPBRIEF + pers_1_2 + LOT_BL_optim +
LOT_BL_pessim + acupuncturist + PROMIS_BL_paininterference.theta

PROMIS_8_physfunct.theta ~ EXPBRIEF + pers_1_2 + LOT_BL_optim +
LOT_BL_pessim + acupuncturist + PROMIS_BL_physfunct.theta

PROMIS_8_sleep.theta ~ EXPBRIEF + pers_1_2 + LOT_BL_optim +
LOT_BL_pessim + acupuncturist + PROMIS_BL_sleep.theta

PROMIS_8_participation.theta ~ EXPBRIEF + pers_1_2 + LOT_BL_optim +
LOT_BL_pessim + acupuncturist + PROMIS_BL_participation.theta

promis_8_29 ~ EXPBRIEF + pers_1_2 + LOT_BL_optim + LOT_BL_pessim +
acupuncturist + promis_1a_29

nrs_8_2 ~ EXPBRIEF + pers_1_2 + LOT_BL_optim + LOT_BL_pessim +
acupuncturist + nrs_1a_2
```

A.2 Baseline With Additional Covariates

```
PROMIS_8_anxiety.theta ~ EXPBRIEF + pers_1_2 + LOT_BL_optim +
LOT_BL_pessim + acupuncturist + age + painduration + CARE_1a_red1 +
PROMIS_BL_anxiety.theta

PROMIS_8_depression.theta ~ EXPBRIEF + pers_1_2 + LOT_BL_optim +
LOT_BL_pessim + acupuncturist + age + painduration + CARE_1a_red1 +
PROMIS_BL_depression.theta

PROMIS_8_fatigue.theta ~ EXPBRIEF + pers_1_2 + LOT_BL_optim +
LOT_BL_pessim + acupuncturist + age + painduration + CARE_1a_red1 +
PROMIS_BL_fatigue.theta

PROMIS_8_paininterference.theta ~ EXPBRIEF + pers_1_2 + LOT_BL_optim +
LOT_BL_pessim + acupuncturist + age + painduration + CARE_1a_red1 +
PROMIS_BL_paininterference.theta
```

```
PROMIS_8_physfunct.theta ~ EXPBRIEF + pers_1_2 + LOT_BL_optim +
LOT_BL_pessim + acupuncturist + age + painduration + CARE_1a_red1 +
PROMIS_BL_ physfunct.theta

PROMIS_8_sleep.theta ~ EXPBRIEF + pers_1_2 + LOT_BL_optim +
LOT_BL_pessim + acupuncturist + age + painduration + CARE_1a_red1 +
PROMIS_BL_sleep.theta

PROMIS_8_participation.theta ~ EXPBRIEF + pers_1_2 + LOT_BL_optim +
LOT_BL_pessim + acupuncturist + age + painduration + CARE_1a_red1 +
PROMIS_BL_ participation.theta

promis_8_29 ~ EXPBRIEF + pers_1_2 + LOT_BL_optim + LOT_BL_pessim +
acupuncturist + age + painduration + CARE_1a_red1 + promis_1a_29

nrs_8_2 ~ EXPBRIEF + pers_1_2 + LOT_BL_optim + LOT_BL_pessim +
acupuncturist + age + painduration + CARE_1a_red1 + nrs_1a_2
```

A.3 Follow-Up With Covariates

```
PROMIS_FU_anxiety.theta ~ EXPBRIEF + pers_1_2 + LOT_BL_optim +
LOT_BL_pessim + acupuncturist + PROMIS_BL_anxiety.theta

PROMIS_FU_depression.theta ~ EXPBRIEF + pers_1_2 + LOT_BL_optim +
LOT_BL_pessim + acupuncturist + PROMIS_BL_depression.theta

PROMIS_FU_fatigue.theta ~ EXPBRIEF + pers_1_2 + LOT_BL_optim +
LOT_BL_pessim + acupuncturist + PROMIS_BL_fatigue.theta

PROMIS_FU_paininterference.theta ~ EXPBRIEF + pers_1_2 + LOT_BL_optim
+ LOT_BL_pessim + acupuncturist + PROMIS_BL_paininterference.theta

PROMIS_FU_physfunct.theta ~ EXPBRIEF + pers_1_2 + LOT_BL_optim +
LOT_BL_pessim + acupuncturist + PROMIS_BL_physfunct.theta

PROMIS_FU_sleep.theta ~ EXPBRIEF + pers_1_2 + LOT_BL_optim +
LOT_BL_pessim + acupuncturist + PROMIS_BL_sleep.theta

PROMIS_FU_participation.theta ~ EXPBRIEF + pers_1_2 + LOT_BL_optim +
LOT_BL_pessim + acupuncturist + PROMIS_BL_participation.theta

promis_fu_29 ~ EXPBRIEF + pers_1_2 + LOT_BL_optim + LOT_BL_pessim +
acupuncturist + promis_1a_29

nrs_fu_2 ~ EXPBRIEF + pers_1_2 + LOT_BL_optim + LOT_BL_pessim +
acupuncturist + nrs_1a_2
```

A.4 Follow-Up With Additional Covariates

```
PROMIS_FU_anxiety.theta ~ EXPBRIEF + pers_1_2 + LOT_BL_optim +
LOT_BL_pessim + acupuncturist + age + painduration + CARE_1a_red1 +
PROMIS_BL_anxiety.theta

PROMIS_FU_depression.theta ~ EXPBRIEF + pers_1_2 + LOT_BL_optim +
LOT_BL_pessim + acupuncturist + age + painduration + CARE_1a_red1 +
PROMIS_BL_depression.theta

PROMIS_FU_fatigue.theta ~ EXPBRIEF + pers_1_2 + LOT_BL_optim +
LOT_BL_pessim + acupuncturist + age + painduration + CARE_1a_red1 +
PROMIS_BL_fatigue.theta

PROMIS_FU_paininterference.theta ~ EXPBRIEF + pers_1_2 +
LOT_BL_optim + LOT_BL_pessim + acupuncturist + age + painduration +
CARE_1a_red1 + PROMIS_BL_paininterference.theta

PROMIS_FU_physfunct.theta ~ EXPBRIEF + pers_1_2 + LOT_BL_optim +
LOT_BL_pessim + acupuncturist + age + painduration + CARE_1a_red1 +
PROMIS_BL_ physfunct.theta

PROMIS_FU_sleep.theta ~ EXPBRIEF + pers_1_2 + LOT_BL_optim +
LOT_BL_pessim + acupuncturist + age + painduration + CARE_1a_red1 +
PROMIS_BL_sleep.theta

PROMIS_FU_participation.theta ~ EXPBRIEF + pers_1_2 + LOT_BL_optim +
LOT_BL_pessim + acupuncturist + age + painduration + CARE_1a_red1 +
PROMIS_BL_ participation.theta

promis_fu_29 ~ EXPBRIEF + pers_1_2 + LOT_BL_optim + LOT_BL_pessim +
acupuncturist + age + painduration + CARE_1a_red1 + promis_1a_29

nrs_fu_2 ~ EXPBRIEF + pers_1_2 + LOT_BL_optim + LOT_BL_pessim +
acupuncturist + age + painduration + CARE_1a_red1 + nrs_1a_2
```

B Glossary of Variable Names

Variable name	Variable explanation	Timepoint	Minimum	Maximum
EAT_BL	Expectation about Acupuncture Treatment Baseline Score	Baseline	5	20
screeningb_1a_4a	pain intensity (at first visit)	Baseline	0	10
nrs_1a_2	pain bothersomeness	Baseline	0	10
painduration	pain duration (at first visit)	Baseline	3	600
LOT_BL_optim	Life Orientation Test - optimism score	Baseline	0	12
LOT_BL_pessim	Life Orientation Test - pessimism score	Baseline	0	12
age	age in years	Baseline	18	65
pers_1_2	gender	Baseline	1 female	2 male
CARE_1a_red1	CARE score (without item 9)	Baseline	8	41
promis_1a_29	PROMIS pain intensity	Baseline	0	10
PROMIS_BL_anxiety.theta	PROMIS Anxiety Theta	Baseline	40.3	81.6
PROMIS_BL_depression.theta	PROMIS Depression Theta	Baseline	41	79.4
PROMIS_BL_fatigue.theta	PROMIS Fatigue Theta	Baseline	33.7	75.8
PROMIS_BL_paininterference.theta	PROMIS Pain interference Theta	Baseline	41.6	75.6
PROMIS_BL_participation.theta	PROMIS Ability to participate to social roles and activities Theta	Baseline	27.5	64.2
PROMIS_BL_physfunct.theta	PROMIS Physical function Theta	Baseline	22.9	56.9
PROMIS_BL_sleep.theta	PROMIS Sleep disturbance Theta	Baseline	32	73.3
PSM_BL	Perceived Sensitivity to Medicines Score	Baseline	5	25
EAT_1b	Expectation about Acupuncture Treatment Score	after the intervention	5	20
EAT_4	Expectation about Acupuncture Treatment Score	after session 4	5	20
nrs_4_1	pain intensity	after session 4	0	10
nrs_8_1	pain intensity	after session 8	0	10
nrs_8_2	pain bothersomeness	after session 8	0	10
promis_8_29	PROMIS pain intensity	after session 8	0	10
PROMIS_8_anxiety.theta	PROMIS Anxiety Theta	after session 8	40.3	81.6
PROMIS_8_fatigue.theta	PROMIS Fatigue Theta	after session 8	41	79.4
PROMIS_8_depression.theta	PROMIS Depression Theta	after session 8	33.7	75.8
PROMIS_8_paininterference.theta	PROMIS Pain interference Theta	after session 8	41.6	75.6
PROMIS_8_participation.theta	PROMIS Ability to participate to social roles and activities zu Basel Theta	after session 8	27.5	64.2
PROMIS_8_physfunct.theta	PROMIS Physical function Theta	after session 8	22.9	56.9
PROMIS_8_sleep.theta	PROMIS Sleep disturbance Theta	after session 8	32	73.3
PSM_8	Perceived Sensitivity to Medicines Score	after session 8	5	25
postguessp_8_1	Post-treatment guess of patients about effectiveness briefing	after session 8	1 regular	2 high
postguessp_8_3	Post-treatment guess of patients about side effect briefing	after session 8	1 regular	2 intensive (detailed)
postguessa_8_1	Post-treatment guess of the acupuncturist about effectiveness briefing	after last session of acupuncture treatment	1 regular	2 high
postguessa_8_3	Post-treatment guess of the acupuncturist about side effect briefing	after last session of acupuncture treatment	1 regular	2 intensive (detailed)
nrs_fu_1	pain intensity	Follow-up	0	10
nrs_fu_2	pain bothersomeness	Follow-up	0	10
promis_fu_29	PROMIS pain intensity	Follow-up	0	10
PROMIS_FU_anxiety.theta	PROMIS Anxiety Theta	Follow-up	40.3	81.6
PROMIS_FU_depression.theta	PROMIS Depression Theta	Follow-up	41	79.4
PROMIS_FU_fatigue.theta	PROMIS Fatigue Theta	Follow-up	33.7	75.8
PROMIS_FU_paininterference.theta	PROMIS Pain interference Theta	Follow-up	41.6	75.6
PROMIS_FU_participation.theta	PROMIS Ability to participate to social roles and activities Theta	Follow-up	27.5	64.2
PROMIS_FU_physfunct.theta	PROMIS Physical function Theta	Follow-up	22.9	56.9
PROMIS_FU_sleep.theta	PROMIS Sleep disturbance Theta	Follow-up	32	73.3
acupuncturist	main acupuncturist	during acupuncture treatment	0 no	1 yes
opioid_use	use of opioids during acupuncture treatment (after inclusion into the study)	during acupuncture treatment		
EXPBRIEF	Effectiveness briefing group			
SIDEBRIEF	Side effect briefing group			

Clinical Study Protocol – other clinical trials

CHANGING PATIENTS' EXPECTATIONS TO INCREASE TREATMENT EFFECTS: A RANDOMIZED CONTROLLED STUDY IN CHRONIC LOW BACK PAIN PATIENTS RECEIVING ACUPUNCTURE

SHORT TITLE (Study Identifier): Pain Acupuncture Treatment Expectation (PATE)

**Study Type:**	Health-related intervention
**Study Categorization:**	Other Clinical Trial Category A
**Study Registration:**	www.clinicaltrials.gov<br>www.kofam.ch
**Study Identifier:**	SNF number 105319_159833 / 1
**Sponsor-Investigator and Principal Investigator:**	Prof. Dr. med. Claudia Witt, MBA<br>Institute for Complementary and Integrative Medicine<br>UniversityHospital Zürich (USZ)<br>Sonneggstrasse 6<br>8091 Zurich<br>Tel: 044 255 23 96<br>Fax: 044 255 43 94<br>Email: claudia.witt@uzh.ch
**Study Intervention:**	Verbal briefing on effectiveness and side effects
**Protocol Version and Date:**	Version 2 , 09.02.2016

CONFIDENTIAL

The information contained in this document is confidential and the property of the Institute of Complementary and Integrative Medicine of the University Hospital Zurich. The information may not - in full or in part - be transmitted, reproduced, published, or disclosed to others than the applicable Independent Ethics Committee(s) and Competent Authority(ies) without prior written authorization from the Institute of Complementary and Integrative Medicine of the University Hospital Zurich, except to the extent necessary to obtain informed consent from those participants who will participate in the study.

SIGNATURE PAGE

Study Title ***CHANGING PATIENTS' EXPECTATIONS TO INCREASE TREATMENT EFFECTS: A RANDOMIZED CONTROLLED STUDY IN CHRONIC LOW BACK PAIN PATIENTS RECEIVING ACUPUNCTURE***

Sponsor-Investigator (Principal Investigator):

This clinical trial protocol was subject to critical review and has been approved by the Sponsor-Investigator. The information herein is consistent with
- the current risk/benefit evaluation of the intervention,
- the moral, ethical and scientific principles governing clinical research as set out in the current version of the Declaration of Helsinki, Good Clinical Practice.

Prof. Dr. med. Claudia Witt

Zürich, 02.02.2016

Place/Date Signature

Biometrician:

Prof. Dr. Leonhard Held

Pfäffikon ZH 6.2.2016

Place/Date Signature

Prof. Dr. Burkhardt Seifert

Zürich, 2.2.2016

Place/Date Signature

TABLE OF CONTENTS

SIGNATURE PAGE 2
STUDY SYNOPSIS 6
LIST OF ABBREVIATIONS 13
STUDY SCHEDULE 14
1 INVESTIGATORS AND STUDY ADMINISTRATIVE STRUCTURE 15
1.1 Sponsor, Sponsor-Investigator (Principal Investigator) 15
1.2 Investigators 16
1.3 Statistician (Biometrician) 16
1.4 Monitoring Institution 17
2 ETHICAL AND REGULATOR ASPECTS 17
2.1 Study Registration 17
2.2 Categorization of the Study 17
2.3 Competent Ethics Committee (CEC) 17
2.4 Ethical Conduct of the Study 18
2.5 Declaration of Interest 18
2.6 Participant Information and Informed Consent 18
2.7 Participant Privacy and Confidentiality 18
2.8 Early Termination of the Study 19
2.9 Protocol Amendments 19
3 INTRODUCTION 19
3.1 Background and Rationale 19
3.2 Study Intervention and Indication 20
3.3 Clinical Evidence to Date 20
3.4 Justification of Study Intervention 21
3.5 Explanation for Choice of Comparator Interventions 21
3.6 Risk/ Benefits 21
3.7 Study Population 22
4 STUDY OBJECTIVES 22
4.1 Overall Objective 22
4.2 Primary Objectives 22
4.3 Secondary Objectives 22
4.4 Safety Objectives 23
5 STUDY OUTCOMES 23
5.1 Primary Outcome 23
5.2 Secondary Outcomes 23
5.3 Additional measures 24
5.4 Safety Outcomes 24
6 STUDY DESIGN AND COURSE OF STUDY 24
6.1 General Study Design and Justification of the Design 24
6.2 Methods of Minimizing Bias 24

6.2.1 Randomization ... *25*
6.2.2 Blinding Procedures ... *25*
6.3 Unblinding Procedures (Code break) ... 25
7 STUDY POPULATION ... 25
7.1 Eligibility Criteria ... 25
7.1.1 Inclusion Criteria ... *25*
7.1.2 Exclusion Criteria ... *25*
7.2 Recruitment and Screening ... 26
7.3 Assignment to Study Groups ... 26
7.4 Criteria for Withdrawal/ Discontinuation of Participants ... 26
8 STUDY INTERVENTION ... 26
8.1 General Information ... 26
8.1.1 Study Intervention ... *28*
8.1.2 Control Intervention ... *28*
8.2 Administration of Study Intervention and Control Intervention ... 28
8.2.1 Study Intervention A) Effectiveness ... *28*
8.2.2 Study Intervention B) Side Effects ... *29*
8.2.3 Control Intervention A) Effectiveness ... *29*
8.2.4 Control Intervention B) Side Effects ... *29*
8.3 Compliance with Intervention ... 29
8.4 Data Collection and Follow-up for Withdrawn Participants ... 29
8.5 Concomitant Intervention(s) ... 29
9 STUDY PROCEDURES ... 30
9.1 Study Flow Chart/Table of Study Procedures and Assessments ... 30
9.2 Assessments of Outcomes ... 30
9.2.1 Assessment of Primary Outcome ... *30*
9.2.2 Assessment of Secondary Outcomes ... *30*
9.2.3 Assessment of Safety Outcomes ... *31*
9.2.4 Assessments in Participants who Prematurely Stop the Study ... *31*
9.3 Procedures at Each Visit ... 32
9.3.1 Screening ... *32*
9.3.2 Baseline (Visit 1a) ... *32*
9.3.3 First acupuncture (Visit 1b) ... *32*
9.3.4 Visit 2 ... *32*
9.3.5 Visit 3 ... *33*
9.3.6 Visit 4 ... *33*
9.3.7 Visit 5-8 ... *33*
9.3.8 End of study (Visit 9) ... *33*
9.3.9 Follow up (Visit 10) ... *33*
10 SAFETY ... 34
10.1 Definitions ... 34
10.2 Recording and Assessment of Serious Adverse Events ... 35
10.3 Reporting of Serious Adverse Events ... 35
10.4 Follow up of (Serious) Adverse Events ... 35

11 STATISTICAL METHODS 36
11.1 Hypotheses 36
11.2 Determination of Sample Size 36
11.3 Planned Analyses 37
11.3.1 Datasets to be Analyzed, Analysis Populations 37
11.3.2 Primary Analysis 37
11.3.3 Secondary Analyses 38
11.3.4 Interim Analyses 38
11.3.5 Safety Analysis 38
11.3.6 Deviation(s) from the Original Statistical Plan 38
11.4 Handling of Missing Data and Drop-Outs 38
12 ELIGIBILITY OF THE PROJECT SITE(S) 38
13 DATA QUALITY ASSURANCE AND CONTROL 39
13.1 DATA HANDLING AND RECORD KEEPING 39
13.1.1 Case Report Forms 39
13.1.2 Specification of Source Documents 40
13.1.3 Record Keeping / Archiving 40
13.2 Data Management 40
13.3 Standard Operating Procedures and internal Monitoring 40
13.4 Confidentiality, Data Protection 41
14 PUBLICATION AND DISSEMINATION POLICY 41
15 FUNDING AND SUPPORT 41
15.1 Funding 41
16 INSURANCE 41
17 REFERENCES 42
18 APPENDICES 44

STUDY SYNOPSIS

**Sponsor / Sponsor-Investigator**	Prof. Dr. med. Claudia Witt, MBA<br>Institute for Complementary and Integrative Medicine<br>UniversityHospital Zurich (USZ)<br>Sonneggstrasse 6<br>8091 Zurich<br>Tel: 044 255 23 96<br>Fax: 044 255 43 94<br>Email: claudia.witt@uzh.ch
**Study Title:**	Changing patients‘ expectations to increase treatment effects: a randomized controlled study in chronic low back pain patients receiving acupuncture
**Short Title / Study ID:**	Pain acupuncture treatment expectation (PATE)
**Protocol Version and Date:**	Version 2, 09.02.2016
**Trial registration:**	www.clinicaltrials.gov<br>www.kofam.ch
**Study category and Rationale**	Other clinical study Category A: the study intervention investigated is associated with very low risk. It entails only minimal risks and burdens.
**Background and Rationale:**	For chronic pain there is a broad consensus about the importance of patient expectations on treatment outcomes (Bialosky, Bishop, & Cleland, 2010); Bishop et al. (2014); (Iles, Davidson, Taylor, & O'Halloran, 2009) Patient expectations are also important for CLBP and acupuncture as shown in secondary analyses of acupuncture trials. However, those analyses do not allow causal conclusions about the importance of expectations on the pathway along the treatment. Experimental study designs with healthy subjects were successful in changing patient expectations and allow causal conclusions. Pain was successfully reduced by verbal suggestions in experimental studies that altered expectations. We now aim to bridge the gap between experimental placebo research and clinical pain research by using verbal suggestions about the possible effects of acupuncture treatment as an intervention to change patients' expectations and influence treatment outcome (i.e. pain). This will also address the research question if expectations mediate pain reduction. In a second research question, our project will study the nocebo response (i.e. reported side effects) in patients receiving intense and detailed information about the possible side effects of acupuncture.

**Objective(s):**	The study wants to manipulate pre-treatment expectations in patients receiving acupuncture and to determine whether the expectation can mediate treatment outcomes. We want to investigate the impact of two verbal briefing interventions, one on effectiveness (placebo) and the other on side effects (nocebo), upon patients' treatment expectation before acupuncture treatment, the experience of side effects during treatment and pain reduction after treatment.
**Outcome(s):**	***Primary outcomes:***<br><br>1a) The primary effectiveness briefing outcome is the expectation of acupuncture treatment (EAT) of patients before their first acupuncture treatment (after the first face-to-face briefing intervention).<br><br>1b) The primary effectiveness outcome after treatment will be the average pain intensity over the last 7 days measured on the numeric rating scale (NRS) (0-10) at 4 weeks, measured.<br><br>2) The primary safety briefing outcome is the side effect score after the total duration of the treatment (4 weeks).<br><br>***Secondary outcomes:***<br><br>- back pain after treatment as measured with the NRS after 2 weeks (short term impact).<br>- back pain bothersomeness measures with the NRS<br>- self-reported health (PROMIS 29) (Cella et al., 2010; Fries, Rose, & Krishnan, 2011; Liu et al., 2010; Rothrock et al., 2010), working alliance (WAI-SR) (Wilmers et al., 2008), Perceived Sensitivity to Medicines (PSM) (Horne et al., 2013)and the amount of pain rescue medication (weekly diary), (Brinkhaus et al., 2006).<br><br>***Additional measures:***<br><br>- Optimism measured with the life orientation test (LOT) (Glaesmer, Hoyer, Klotsche, & Herzberg, 2008).<br>- Empathy with the doctor who performs the briefing using the German Version of the "Consultation and Relational Empathy" (CARE) instrument (Neumann et al., 2008).<br><br>***Safety***: SAE occurrence and side effects of acupuncture (primary outcome)
**Study design:**	We will perform a randomized double-blind four-armed trial with a 2 x 2 factorial design (factors: effectiveness briefing (regular vs. high) and side effect briefing (regular vs. intense)) with a four-week treatment per patient.

**Inclusion / Exclusion criteria:**	Inclusion criteria:<br>Male and female patients aged 18 to 65 years with chronic non-specific low back pain (CLBP) according to Deyo et al. (2014) with an average pain severity of at least 4 on a 0-10 numeric rating scale (NRS) in the last 7 days and with pain in at least half of the days per month in the last 6 months will be eligible for this study.<br><br>Exclusion criteria:<br>Patients will be excluded if they had have acupuncture treatment within the last 12 months; are breast-feeding or are known pregnant, have a known blood coagulation disorder or intake of coagulation inhibiting drugs with the exception of aspirin. Furthermore they are excluded in case of their CLBP being caused by a known malignant disease, trauma, the presence of a known rheumatic or autoimmune disorder, a history or planned surgery of the spinal column of the low back in the next 6 months, regular intake of analgesics (> once per week) because of additional disease, intake of centrally-acting analgesics, regular intake of corticosteroids or injections with corticosteroids within the last 8 weeks before randomization (specified in a studyspecific SOP), or a history of severe acute or chronic disorders that do not allow participation in the therapy. Further exclusion criteria include known alcohol or substance abuse, insufficient German language skills, current application for a pension claim.

**Study Intervention:**	**Effectiveness:**<br>The goal of this briefing is to change the patient's expectation about his or her clinical outcome of their acupuncture.<br>The high expectation briefing will emphasize the large overall effects of acupuncture, which have been demonstrated in many studies, and highlight that acupuncture was superior to conventional standard care for CLBP. Patients will be told that, since acupuncture requires extensive training, the acupuncture therapists have received special training and are under close supervision of other team members so that they can provide a very high quality treatment. Patients will also receive the information that our team was very successful in using acupuncture to treat similar patients with CLBP, and we have shown the effectiveness of acupuncture in large clinical trials. Finally, patients will be informed that most patients are very satisfied with acupuncture treatment and would recommend it to other patients.<br>The main study intervention will be performed before the first acupuncture session (duration around 30 minutes). At the second visit, a face-to-face booster session will take place (duration around 15 minutes). Furthermore, patients will receive a booster email or letter after the third and sixth acupuncture session including a short resume concerning the effectiveness of acupuncture and more information on the side effects that could occur.<br><br>**Side effects:**<br>The goal of the intense safety briefing is to provide information sensitizing patients to the presence of possible side effects during acupuncture treatment. The doctor will explain in detail the side effects that have been observed in a large study with over 2 million acupuncture treatments. Patients will receive the patient information leaflet that was developed based on this study and the doctor will advise them to read it carefully because he would like to discuss any questions they may have during the next visit.<br>During the booster sessions the medical doctor will ask if side effects have occurred, explain the possible side effects again, and ask the patients if they have any questions about side effects.

**Reference Intervention:**	**Effectiveness:**<br>In the regular expectation briefing it will be mentioned that acupuncture may help to overcome CLBP. However, since CLBP is a chronic condition, patients have to be realistic about treatment benefits. Only about half of the patients will benefit from acupuncture and professionals are unable to decide in advance if acupuncture will be beneficial for a specific patient. It will also be outlined and explained that sham acupuncture has a comparably high success rate.<br>The reference intervention of the study will be performed before the first acupuncture session (duration around 30 minutes). Furthermore, patients will receive an email or letter after the third and sixth acupuncture session including the reminder of the appointments made. It will include a short resume concerning acupuncture but will not mention any side effects.<br><br>**Side effects:**<br>A regular briefing about side effects is part of the informed consent and will be delivered to all four groups. The regular safety briefing means that no additional information about side effects will be provided in the briefing sessions.
**Medical treatment**	All patients will receive a standardized acupuncture which consists of 6 points that are not typical acupuncture points, but have been shown to be effective in previous trials (resulted in a 50% pain severity reduction in 38% of the patients).<br>8 sessions over 4 weeks (2 times per week) will be provided. The needles used are CE certified.
**Number of Participants with Rationale:**	128 patients (64 high expectation briefing and 64 regular expectation briefing as well as 64 intense side effect briefing and 64 regular side effect briefing) will be needed.<br>Assuming a dropout rate of approximately 15%, we plan to enroll 150 patients.
**Study Duration:**	Data collection (recruitment to LPLV) will start in March 2016 and take around 18 months (i.e., until September 2017).
**Study Schedule:**	FPFV March 2016<br>LPLV December 2017

**Investigator(s):**	PD Dr. phil. Jürgen Barth<br>Institute for Complementary and Integrative Medicine<br>University Hospital Zurich<br>Sonneggstrasse 6<br>8091 Zurich<br>juergen.barth@usz.ch<br>+41 44 255 48 96<br><br>Prof. Dr. med. Thomas Rosemann, PhD<br>Institute for Family Medicine<br>University Hospital Zurich<br>Pestalozzistrasse 24<br>8091 Zurich<br>thomas.rosemann@usz.ch<br>+41 44 255 90 99<br><br>Prof. Dr. Leonhard Held<br>University of Zurich<br>Epidemiology, Biostatistics and Prevention Institute (EBPI)<br>Hirschengraben 84<br>8001 Zurich<br>leonhard.held@uzh.ch<br>+41 44 63 44640<br><br>Prof. Dr. Burkhardt Seifert<br>University of Zurich<br>Epidemiology, Biostatistics and Prevention Institute (EBPI)<br>Hirschengraben 84<br>8001 Zurich<br>seifert@ifspm.uzh.ch<br>+41 44 63 44644<br><br>Dr. Maren Cordi<br>Institute for Complementary and Integrative Medicine<br>Sonneggstrasse 6<br>8091 Zurich<br>maren.cordi@usz.ch<br>+41 44 255 94 51<br><br>Anja Zieger<br>Institute for Complementary and Integrative Medicine<br>Sonneggstrasse 6<br>8091 Zurich<br>Anja.zieger@usz.ch<br>+41 44 255 50 46<br><br>Alexandra Kern<br>Institute for Complementary and Integrative Medicine<br>Sonneggstrasse 6<br>8091 Zurich<br>Alexandra.kern@usz.ch<br>+41 44 255 51 45

**Study Centre(s):**	Unicentric:<br>Institute for Complementary and Integrative Medicine<br>Sonneggstrasse 6<br>8091 Zurich
**Statistical Considerations:**	We will use an analysis of covariance (ANCOVA) with the fixed effects briefing group and sex and the covariate baseline expectation (1a); the fixed effects briefing group, sex, therapist and the covariates baseline pain and optimism (1b).<br>For research question 2, zero-inflated and hurdle models for count data will be used.<br>Research question 3 will be analyzed using regression analyses with baseline back pain severity, age, and sex as covariates.
**GCP Statement:**	This study will be conducted in compliance with the protocol, the current version of the Declaration of Helsinki, the ICH-GCP as well as all national legal and regulatory requirements.

LIST OF ABBREVIATIONS

ClinO	Clinical Trial Ordinance (KlinV)
CRF	Case Report Form
GCP	Good Clinical Practice
ICH	International Conference on Harmonization
PI	Principal Investigator
SAE	Serious Adverse Event
SOP	Standard Operating Procedure
TCM	Traditional Chinese Medicine
CLBP	Chronic Low Back Pain
NRS	Numeric Rating Scale
WAI-SR	Working Alliance Inventory (short revised)
PROMIS	Patient-Reported Outcomes Measurement Information System
PSM	Perceived Sensitivity to Medicines
CARE	Consultation and Relational Empathy
LOT	Life Orientation Test
EAT	Expectation of Acupuncture Treatment

STUDY SCHEDULE

**Period**	**Scree-ning** (phone)	**Base-line** (Briefer)	**Acupuncture treatment (acupuncturist, patient)**									**Fol-low-up mon-th 6**	**Stud y end**
**Visit**	**scree ning**	**1a**	**1b**	**2**	**3**	**4**	**5**	**6**	**7**	**8**		**9**	**10**
Patientinfor-mation and consent form	S4	B2											B6
Soziodemo-graphic data	S1	B1											
Inclusion and exclusion criteria	S1	B1											
Patient history /comorbidity		B1											
Appointment sheet	S3	S3											
Randomi-zation		B1											
Adherence physician		B4		B5									
Adherence therapist			A1	A2	A2	A2	A2	A2	A2	A2			
Pain (NRS)	S1	B1, P1				P7				P7		P12	
Expectation acupuncture (EAT)		P1, P2				P2							
Bothersome-ness (NRS)		P1				P7				P7		P12	

Optimism (LOT)		P1											
Health (PROMIS 29)		P1				P3				P3		P12	
Empathy (CARE)		P4											
Working Alliance Inventory (WAI-SR)			P5							P5			
Perceived Sensitivity to Medicines Scale (PSM)		P1								P8			
Post-treat-ment Guess										A3, P10			
Side effects				P9	P9	P9	P9	P9	P9	P9			
Rescue medication			P6	P6	P6	P6	P6	P6	P6	P6			
Dropout questionnaire P11*													

* after dropout

1 INVESTIGATORS AND STUDY ADMINISTRATIVE STRUCTURE

1.1 Sponsor, Sponsor-Investigator (Principal Investigator)

Name: Prof. Dr. med. Claudia Witt, MBA
Adress: Institute for Complementary and Integrative Medicine
UniversityHospital Zurich
Sonneggstrasse 6, 8091 Zurich
Email: claudia.witt@uzh.ch
Tel: +41 44 255 23 96
Fax: +41 44 255 43 94

1.2 Investigators

PD Dr. phil. Jürgen Barth
Institute for Complementary and Integrative Medicine
University Hospital Zurich
Sonneggstrasse 6
8091 Zurich
juergen.barth@usz.ch
+41 44 255 48 96

Prof. Dr. med. Thomas Rosemann, PhD
Institute for Family Medicine
University Hospital Zurich
Pestalozzistrasse 24
8091 Zürich
thomas.rosemann@usz.ch
+41 44 255 90 99

Dr. Maren Cordi
Institute for Complementary and Integrative Medicine
Sonneggstrasse 6
8091 Zurich
maren.cordi@usz.ch
+41 44 255 94 51

Anja Zieger
Institute for Complementary and Integrative Medicine
Sonneggstrasse 6
8091 Zurich
Anja.zieger@usz.ch
+41 44 255 50 46

Alexandra Kern
Institute for Complementary and Integrative Medicine
Sonneggstrasse 6
8091 Zurich
Alexandra.kern@usz.ch
+41 44 255 51 45

1.3 Statistician (Biometrician)

Prof. Dr. Leonhard Held
University of Zurich
Epidemiology, Biostatistics and Prevention Institute (EBPI)
Hirschengraben 84
8001 Zurich
leonhard.held@uzh.ch

+41 44 63 44640

Prof. Dr. Burkhardt Seifert
University of Zurich
Epidemiology, Biostatistics and Prevention Institute (EBPI)
Hirschengraben 84
8001 Zurich
seifert@ifspm.uzh.ch
+41 44 63 44644

1.4 Monitoring Institution

Due to the low risk associated with the Category A study, the Monitoring will be done internally by members of the Institute for Complementary and Integrative Medicine, UniversityHospital Zurich, Zurich, Switzerland who have GCP courses 1&2 and are not further involved in the study.

2 ETHICAL AND REGULATOR ASPECTS

Before this study will be conducted, the protocol, the proposed participant information and consent form as well as other study-specific documents will be submitted to a properly constituted Competent Ethics Committee (CEC) in agreement with local legal requirements, for formal approval.

The decision of the CEC concerning the conduct of the study will be made in writing to the Sponsor-Investigator before commencement of this study. The clinical study can only begin once approval from the CEC has been received.

2.1 Study Registration

This trial will be registered in the coordination office for research on humans (Kofam), the Swiss Federal Complementary Database and the international trial registry ClinicalTrials.gov (clinicaltrials.gov).

2.2 Categorization of the Study

Category A: the study intervention investigated is associated with very low risk. It entails only minimal risks and burdens.

2.3 Competent Ethics Committee (CEC)

Approval from the appropriate constituted Competent Ethics Committee is sought for the clinical trial. The reporting duties and allowed time frame are respected. No substantial amendments are made to the protocol without prior CEC approval, except where necessary to eliminate apparent immediate hazards to study participants.

Premature study end or interruption of the study is reported within 15 days. The regular end of the study is reported to the CEC within 90 days, the final study report shall be submitted within one year after study end. Amendments are reported according to chapter 2.9.

2.4 Ethical Conduct of the Study

The study will be carried out in accordance with principles enunciated in the current version of the Declaration of Helsinki, the guidelines of Good Clinical Practice (GCP) issued by ICH, and Swiss competent authority's requirements.

CEC will receive annual safety and interim reports and be informed about non-substantial amendments, the course of the study, and the study stop/ end in agreement with local requirements.

2.5 Declaration of Interest

We declare that there are no conflicts of interest.

2.6 Participant Information and Informed Consent

The investigator must explain to each participant the nature of the study, its purpose, the procedures involved, the expected duration, the potential risks and benefits and any discomfort it may entail. Each participant must be informed that the participation in the study is voluntary and that he/she may withdraw from the study at any time and that withdrawal of consent will not affect his/her subsequent medical treatment.

The participant must be informed that his/her medical records may be examined by authorized individuals other than their treating physician.

All Participants for this study will be provided a participant information sheet and a consent form describing this study and providing sufficient information for participants to make an informed decision about their participation in this study.

The participant information sheet and the consent form will be submitted with the protocol for review and approval for the study by the CEC. The formal consent of a participant, using the approved consent form, must be obtained before that participant is submitted to any study procedure.

The participant should read and consider the statement before signing and dating the informed consent form, and should be given a copy of the signed document. The consent form must also be signed and dated by the investigator (or his designee) and it will be retained as part of the study records.

2.7 Participant Privacy and Confidentiality

The investigators are liable to treat the entire information related to the study and the compiled data strictly confidentially. Any passing-on of information to persons that are not directly involved in the study must be approved by the owner of the information.

Data generation, transmission, archiving and analysis of personal data within this study, strictly follows the current Swiss legal requirements for data protection. Prerequisite is the voluntary approval of the Participant given by signing the informed consent prior start of participation of the clinical trial.

Individual participant medical information obtained as a result of this study is considered confidential and disclosure to third parties is prohibited. Participant's confidentiality will be further ensured by utilizing participant identification code numbers to correspond to treatment data in the computer files.

Such medical information may be given to the participant's personal physician or to other appropriate medical personnel responsible for the participant's welfare, if the patient has given his/her written consent to do so.

Data generated as a result of this study are to be available for inspection on request by the monitors and by the CEC.

2.8 Early Termination of the Study

The Sponsor-Investigator may discontinue the study prematurely according to certain circumstances:

- ethical concerns,
- insufficient participant recruitment.

2.9 Protocol Amendments

Substantial amendments are only implemented after approval of the CEC.
Under emergency circumstances, deviations from the protocol to protect the rights, safety and well-being of human participants may proceed without prior approval of the sponsor and the CEC. Such deviations shall be documented and reported to the sponsor and the CEC as soon as possible.
All Non-substantial amendments are communicated to the CEC within the Annual Safety Report (ASR).

3 INTRODUCTION

3.1 Background and Rationale

Chronic low back pain (CLBP) is a highly prevalent medical condition and CLBP patients often use acupuncture (Deglon-Fischer, Barth, & Ausfeld-Hafter, 2009; Hoy et al., 2012). That acupuncture reduces chronic pain substantially has been shown in clinical trials and meta-analyses (e.g., (Berman, Langevin, Witt, & Dubner, 2010). About half of the effect can be explained by non-specific effects including those induced by expectation. This study wants to investigate the impact of two verbal briefing interventions, one on effectiveness (placebo) and the other on side effects (nocebo), upon patients' treatment expectation before their first acupuncture, experience of side effects during treatment and pain reduction after treatment. The study wants to manipulate pre-treatment expectations in patients receiving sham acupuncture and to determine whether the expectation can mediate treatment outcomes. It thereby builds on evidence from previous research and uses a translational approach by bridging between results from experimental placebo research and clinical trials on acupuncture. If it turns out that a briefing intervention about effectiveness can increase expectation and that expectations mediate treatment effects such a briefing intervention could be implemented into primary care to improve patients' outcomes. Furthermore, the research model might be used for other diseases or other briefing interventions.

For chronic pain there is a broad consensus about the importance of patient expectations on treatment outcomes (Bialosky et al., 2010; Bishop et al., 2014; Iles et al., 2009). Patient expectations are also important for CLBP and acupuncture as shown in secondary analyses of acupuncture trials. However, those analyses do not allow causal conclusions about the importance of expectations on the pathway along the treatment. Experimental study designs with healthy subjects were successful in changing patient expectations and allow causal conclusions. Pain was successfully reduced by verbal suggestions in experimental studies that altered expectations. Therefore, we want to move the field forward with a study that allows applying an experimental methodology in a clinical setting. This goes along with the suggested implication for research of Bialosky and colleagues: "Further longitudinal studies specific to the experimental manipulation of expectation are needed in clinical samples […]." (Bialosky et al., 2010). We aim to bridge the gap between experimental placebo research and clinical pain research by using verbal suggestions about the possible effects of acupuncture treatment as an intervention to change patients' expectations and influence treatment outcome (i.e. pain). This will also address the research question if expectations mediate pain reduction. In a second research question, our project will study the nocebo response (i.e. reported side effects) in patients receiving intense and detailed information about the possible side effects of acupuncture.

3.2 Study Intervention and Indication

The study wants to manipulate pre-treatment expectations in patients receiving acupuncture and to determine whether the expectation can mediate treatment outcomes. The impact of two verbal briefing interventions, one on effectiveness (placebo) and the other on side effects (nocebo) will be investigated. Briefing for treatment expectations will be varied between high and regular; briefing for side effects will be varied between intense and regular.

3.3 Clinical Evidence to Date

Expectation:
Research shows that expectations of clinical improvements in pain play the most important role for the placebo response (Enck, Benedetti, & Schedlowski, 2008). Larger expected benefits of a treatment go along with larger effects of treatments across different medical conditions (Mondloch, Cole, & Frank, 2001).
In cohort studies, patients' positive expectations have been shown to decrease pain after medical treatment (Bialosky et al., 2010; Bishop et al., 2014; Iles et al., 2009). Patient expectations also affect the acupuncture treatment effect. A pooled analysis of our previous acupuncture trials including more than 800 patients with chronic pain showed that patients' expectations predicted acupuncture treatment response (Linde et al., 2007). Patients had higher odds (odds ratio 2.11 [CI 1.32 – 3.34]) of benefitting from acupuncture if they expected a better treatment response before the intervention. This finding holds for regular acupuncture as well as for sham acupuncture.

Side effects:
Research has shown that when compared to a focus on treatment risks, no information or positive framing of side effects results in fewer side effects (O'Connor, Pennie, & Dales, 1996; Varelmann, Pancaro, Cappiello, & Camann, 2010).

Acupuncture:
Acupuncture has been demonstrated to be an effective treatment for CLBP (Berman et al., 2010). Our recent patient-level data meta-analyses found for CLBP and neck pain a clinically relevant effect (effect size .55) for acupuncture compared to active control treatments (Vickers

et al., 2012). However, the difference between acupuncture and sham acupuncture was statistically significant, but of small size (effect size .23). This shows that a substantial proportion of patients experience clinically relevant pain relief under sham acupuncture.

3.4 Justification of Study Intervention

In experimental placebo research, so-called “verbal suggestions” provided as information to the participants have been widely used to change participants’ expectations about the effects of a placebo drug. In clinical studies, the information patients receive about treatments and their efficacy before the treatment have an impact on effects (Crow et al., 1999; Kirsch, 1997).
It is common in some disciplines to incorporate interventions that strengthen patients’ expectations about treatment effects into the treatment itself. In cognitive behavioral therapy patients with a motivational interviewing intervention in addition to their psychological treatment showed larger improvements and greater treatment compliance (Westra & Dozois, 2006). Such an intervention strategy can also be used in complementary medicine. Clinicians using complementary medicine reported four different strategies to enhance patients’ expectations: creating hope, conditional prognosis, expressing confidence, and promotion of patients’ self-efficacy (Dellmann & Lushington, 2012). However, this research in complementary medicine is more on an observational or narrative level, but experimental studies on how to change expectations or links to current evidence from experimental placebo research are missing.

3.5 Explanation for Choice of Comparator Interventions

To be able to observe if expectation can be changed by different briefings and if this can affect treatment outcome, we plan to compare a high expectation briefing with a regular expectation briefing. We use this approach for both research questions, effectiveness and side effects within this 2x2 factorial design. The content of the briefings is associated with how it could be commonly used in usual care depending on the current practice of different clinicians. To induce a clinical relevant effect size we decided to include booster session to increase the possible effect and reduce the sample size.

3.6 Risk/ Benefits

Risks associated with the briefing intervention:
The experimental manipulation of expectations by the briefing intervention will not be mentioned in advance since the study can only explore this effect without disclosing this study aim to patients. Blinding patients about the use of different briefing interventions is essential to get a valid response. The study objectives will be fully explained to the patients after the end of the study (LPLV), which will be after the last acupuncture session and the last measurement of the study outcomes. Patients will be asked for a full retrospective informed consent. If they consent, data about effectiveness and side effects will be used in the analysis. In case of a refused consent data about effects and side effects will not be used. In this case, only basic information from the screening will be used to describe patients who have withdrawn consent. Such a procedure is covered by the legal situation in Switzerland (Schweizerischer Bundesrat, 2009).

The briefing interventions are developed to make sure that ethically problematic situations are avoided. Patients will not be discouraged or demotivated in the regular briefing condition. This briefing condition is quite similar to patient information before regular treatment. Also the condition with a detailed description of side effects will not differ much from the other empirical investigations on acupuncture. The only difference between the information that will be given in the study and regular patient information in daily practice is the use of booster sessions. This

additional booster is necessary to have a stronger briefing intervention. A less strong intervention might have less effect and would require more patients. In order to minimize effort for a larger number of patients a more intensive briefing intervention is necessary.

Risks associated with acupuncture:
All patients will receive an acupuncture treatment, which is a highly effective treatment for CLBP. The effectiveness of our acupuncture treatment is only slightly lower than for the one for a typical Chinese acupuncture which follows the concept of TCM (Traditional Chinese Medicine) and was superior to standard care in a study. Patients also receive the information that they are allowed to use rescue pain medication and they are allowed to seek additional medical support if needed.

3.7 Study Population

Male and female patients aged 18 to 65 years with CLBP averaging a severity of at least 4 (on a 0-10 point numeric rating scale, NRS (Deyo et al., 2014) in the last seven days will be eligible for this study. Patients with low back pain longer than 3 months in duration and present for at least half the days in the past 6 months will be classified as having CLBP.

4 STUDY OBJECTIVES

4.1 Overall Objective

The study wants to manipulate pre-treatment expectations in patients receiving acupuncture and to determine whether the expectation can mediate treatment outcomes. This study is building on evidence from previous research and uses a translational approach by bridging between results from experimental placebo research and clinical trials on acupuncture. If it turns out that a briefing intervention about effectiveness can increase expectation and that expectations mediate treatment effects, such a briefing intervention could be implemented into primary care to improve patients' outcomes. Furthermore, the research model might be used for other diseases or other briefing interventions.

4.2 Primary Objectives

We want to investigate the impact of two verbal briefing interventions, one on effectiveness (placebo) and the other on side effects (nocebo), upon patients' treatment expectation before acupuncture treatment, the experience of side effects during treatment and pain reduction after treatment.

4.3 Secondary Objectives

Secondary aims of this study include evaluating the impact of the effectiveness briefing on back pain bothersomeness, self-reported health (PROMIS), working alliance of the acupuncturist (WAI-SR), the perceived sensitivity to medicines (PSM) and the use of pain rescue medication (measured in a weekly diary) at several time points.

Furthermore we will evaluate optimism (LOT), the empathy towards the physician (CARE) and perform a post-treatment guess to check whether treatment providers and patients stayed blinded to briefing assignments.

4.4 Safety Objectives

We will investigate the occurrence of SAE as well as assess acupuncture side effects, which are a primary objective.

5 STUDY OUTCOMES

5.1 Primary Outcome

We have two independent research questions (hypotheses 1 and 2) and overall three primary outcomes for these questions.

The primary effectiveness briefing outcome is the expectation of acupuncture treatment (EAT) of patients before their first acupuncture treatment (after the first face-to-face briefing intervention). Patient expectations will be assessed by a self-report measure using a Likert scale and a numeric rating scale. The final EAT score can range from 1 to 10.

A second primary outcome is back pain severity, which will be assessed by a numeric rating scale, measuring the average back pain intensity over the last 7 days after 4 weeks.

The primary safety briefing outcome is the side effect score. Side effects of the last treatment will be documented before each treatment (=adverse effects) and for each reported side effect patients will be asked to rate the intensity on a scale from 1-3 (low, moderate, strong). The intensity numbers will be summarized in a side effect score. We assess the side effects in order to measure the effect of the nocebo briefing.

In the statistics section is described how the multiple primary endpoints are handled to rule out multiple testing.

5.2 Secondary Outcomes

The secondary outcomes of this study are back pain severity at 2 weeks after treatment, measured with the numeric rating scale (NRS 0-10), to have a short term measure, the back pain bothersomeness measured with an NRS (0-10), self-reported health as measured with the PROMIS 29 (Patient-Reported Outcomes Measurement Information System) (Cella et al., 2010; Fries et al., 2011; Liu et al., 2010; Rothrock et al., 2010) as well as the use of pain rescue medication to accumulate health data (measured with a weekly diary) (Brinkhaus et al., 2006).
We assess the alliance of the acupuncturist (WAI-SR), (Wilmers et al., 2008) to control for possible influences of the acupuncturists. Lastly, we measure the perceived sensitivity to medicines (PSM), (Horne et al., 2013).

5.3 Additional measures

At baseline, we measure the optimism with the Life-orientation Test (LOT-R, (Glaesmer et al., 2008)). After completion of treatment, acupuncture therapists will be asked to guess which interventions patients received and their confidence with this guess. This will allow checking whether treatment providers were successfully blinded to briefing assignments. Patients will be also asked to guess which briefing interventions they received. The patient guesses will be obtained after disclosure of the study aim at the end of the treatment.

In addition we measure the empathy of the physician who performs the briefing, using the German Version of the “Consultation and Relational Empathy“ (CARE) instrument (Neumann et al., 2008).

5.4 Safety Outcomes

The primary safety measure is the patient report regarding SAEs which will be documented in the SAE report form. Furthermore we will assess acupuncture side effects, which are defined as a primary outcome (please see above).

STUDY DESIGN AND COURSE OF STUDY

6.1 General Study Design and Justification of the Design

We will perform a randomized double-blind four-armed trial with a 2 x 2 factorial design with two independent factors (effectiveness briefing and side effect briefing). All patients will have a four-week acupuncture treatment (8 sessions) and receive a follow up questionnaire after 6 months. Before the first acupuncture session (ca. 30 minutes) and before the second acupuncture session (ca. 15 minutes), there will be a consultation with the physician in which the briefing interventions will take place. We aim to recruit a total of 150 patients.

We will use central block-randomization with variable block length in a 1:1:1:1 ratio.

We will test the following null hypotheses:
Research question on effectiveness:
(1a) high expectation briefing = regular expectation briefing for change in expectation
(1b) high expectation briefing = regular expectation briefing for change in back pain
a priori ordered hierarchical test procedure will be used
Research question on side effects:
(2) high side effect briefing = regular side effect briefing for amount of side effects reported.

6.2 Methods of Minimizing Bias

To reduce bias, we randomize the patients to the intervention. Patients in the study will not be aware that briefing is the study intervention and they will be blinded to the briefing group application. The allocation is concealed by using the software secuTrial®. We will furthermore ask the patients to provide a post-treatment guess to measure whether blinding patients was successful. The acupuncturists will be blinded and we will ask them for a post-treatment guess as well.

6.2.1 Randomization

We will use central block-randomization with variable block length in a 1:1:1:1 ratio. Randomization sequence will be stratified by sex using R (Version 3.1.0) by a statistician not further involved in the study. For allocation of patients via the internet the software secuTrial® (Clinical Trial Center, University Hospital Zurich) will be used (see Appendix).

6.2.2 Blinding Procedures

We will perform a randomized double-blind design: patients, and acupuncture therapists will be blinded to patient group assignment. The PhD students will screen patients for study inclusion and obtain informed consent. A medical doctor not involved in the acupuncture treatment will receive the randomization assignment for the patient and carry out the briefing interventions. The acupuncture therapists do not have any information about the allocation of patients. The patients do not have any information about the study aim and will be asked for a retrospective consent after the end of the study. Thus, it is not expected that participants know about any expectancy and side effect manipulation and hence will not realize the difference between study intervention and control intervention.

Towards the public we have to blind the study aim and therefore communicate the study aim as agreed with the SNF Forschungsrat: “we aim to develop an instrument for assessing patient’s expectations, implement it practically and test the influence of expectations on treatment outcome.”

6.3 Unblinding Procedures (Code break)

Not intended because all patients receive the same medical treatment.

7 STUDY POPULATION

7.1 Eligibility Criteria

7.1.1 Inclusion Criteria

Male and female patients aged 18 to 65 years with chronic non-specific low back pain (CLBP) according to Deyo et al. (2014) with an average pain severity of at least 4 on a 0-10 numeric rating scale and at least half of the days per months with pain in the last 6 months will be eligible for this study.

7.1.2 Exclusion Criteria

Patients will be excluded if they had have acupuncture treatment within the last 12 months; are breast-feeding or are known pregnant, have a known blood coagulation disorder or take of coagulation inhibiting drugs with the exception of aspirin. Furthermore they are excluded in case of their CLBP being caused by a known malignant disease, trauma, the presence of a known rheumatic or autoimmune disorder, a history or planned surgery of the spinal column of the low back in the next 6 months, regular intake of analgesics (> once per week) because of additional disease, intake of centrally-acting analgesics, regular intake of corticosteroids or injections with corticosteroids within the last 8 weeks before randomization (specified in a studyspecific SOP),

or a history of severe acute or chronic disorders that do not allow participation in the therapy. Further exclusion criteria include known alcohol or substance abuse, insufficient German language skills, current application for a pension claim.

7.2 Recruitment and Screening

Patients will be recruited via family physicians from a research network. This research network is managed by the Institute of Family Medicine of the University Zurich and includes 200 physicians that are used to contribute to research. The family physician research network is currently not involved in any other research projects on CLBP that may interfere with the recruitment for this trial. Furthermore, the Horten Center at the University Zurich hosts a comprehensive register of low back pain patients and will inform about the study. In addition to these recruitment strategies, patients will be recruited via local newspapers, patient organizations, and via the website of the Institute for Complementary and Integrative Medicine. The recruitment of about 150 patients with CLBP during a period of 18 months seems well feasible. For recruitment, residents who perform under the supervision of Prof. Rosemann will be asked to inform their chronic low back pain patients in the outpatient clinics about the possibility to take part in our study. For ensuring strong and reliable support, we will build up a close network to those doctors.

As a second recruitment strategy, we will contact company doctors through the Swiss association of occupational medicine and ask them for their assistance in making our study public to their patients.

Patients will not be financially compensated for their participation.

7.3 Assignment to Study Groups

Randomization will be conducted by the doctor who includes the patients into the study using the software secuTrial® (Clinical Trials Center Zurich), to ensure allocation concealment (see Appendix).

7.4 Criteria for Withdrawal/ Discontinuation of Participants

Participants have the right to abort the study at any time and without providing a reason. The study coordinator has the right to exclude participants if they provide wrong information or do not follow the safety guidelines.

8 STUDY INTERVENTION

8.1 General Information

Study interventions (briefing intervention)
Group 1: High expectation briefing + regular briefing about side effects (no additional briefing)
Group 2: High expectation briefing + intense briefing about side effects (additional briefing)

Group 3: Regular expectation briefing + regular briefing about side effects (no additional briefing)
Group 4: Regular expectation briefing + intense briefing about side effects (additional briefing)

Procedure:

Independent of the briefing intervention all patients will receive the patient information leaflet about the study with common information about possible side effects as part of the informed consent procedure at the first visit. We transferred the basic safety profile from a study observing over 2 million acupuncture treatments into a patient information leaflet that is available for routine clinical care (Witt et al., 2009).

The briefing interventions will be provided through in-person contact with the doctor and also include written materials for the patients to take home before the first treatment session. The written materials have been adapted to the four groups. The initial effectiveness briefing will have a duration of about 20 minutes; the initial safety briefing will have a duration of 10 minutes. A manual for the briefing interventions has been developed and the medical doctors providing the briefing intervention will be trained in the briefing intervention as well as in communication skills (empathic style).
Patients will have a face-to-face booster visit according to their group allocation at the second treatment session, which will take around 15 minutes (10 minutes effectiveness briefing and 5 minutes safety briefing).

As a second booster, patients will receive emails or letters including a reminder of their appointments, more information about acupuncture for chronic low back pain as well as possible side effects according to their group allocation after the third and sixth acupuncture session.

Medical treatment

All patients will receive a standardized acupuncture. Acupuncture has been used successfully before in trials applying verbal suggestion as an intervention, and has been shown to be superior to conventional standard care (Haake et al., 2007). All patients will receive the same penetrating acupuncture that is provided at no acupuncture points (6 predefined points bilateral). It has been developed in a consensus procedure for our previous trial and resulted in a 50% pain severity reduction in 38% of the patients (Brinkhaus et al., 2003; Brinkhaus et al., 2006). Patients in this trial will receive 8 sessions (two per week) over four weeks, because one of our recent trials found > 25mm pain reduction on a VAS after 8 treatments (Pach et al., 2013) and most trials in our patient level meta analyses provided between 6 and 10 treatments (MacPherson et al., 2013). Needles (B type needles, CE-certified, sterile and in single use, size No.8 (0.30) x 30mm, produced by medical Seirin Corporation, Shizuoka, Japan) will be left in for 25 minutes and will be manually stimulated twice (beginning and end of the treatment sessions). Stimulation will be standardized.
According to the communication and the request by the SNF Forschungsrat, we will call this type of medical treatment acupuncture or scientific effective acupuncture, in German „wissenschaftlich wirksame Akupunktur“.
The first acupuncture treatment will be delivered after the briefing by two therapists (PhD students) that will be especially trained for this type of acupuncture. Previous experience shows that this type of standardized acupuncture is easy to learn and to apply. Acupuncture training will include video documentation, extensive hands on training and supervision by a medical doctor with experience in this type of acupuncture. The patient-therapist interaction will be standardized and performed in the same treatment rooms for all patients. We have developed a manual for the communication style and will train the acupuncture therapists. The therapists will be especially trained to perform this standardized patient-doctor interaction in order to limit

biases due to this interaction. Therapists will document the adherence to the acupuncture treatment and to the standardized patient-doctor interaction after each session. All deviations from the study protocol will be documented. Since the therapists have to deliver the same treatment for all experimental conditions the study team is very optimistic about delivering a highly standardized treatment. Supervision will be provided by the principal investigator.

8.1.1 Study Intervention

Study Intervention A) Effectiveness

Briefing intervention: high expectation

Study Intervention B) Side Effects

Briefing intervention: intense briefing about side effect

8.1.2 Control Intervention

Control Intervention A) Effectiveness

Briefing intervention: regular expectation

Control Intervention B) Side Effects

Briefing intervention: regular briefing about side effects

8.2 Administration of Study Intervention and Control Intervention

8.2.1 Study Intervention A) Effectiveness

The *high expectation briefing* will emphasize the large overall effects of acupuncture, which have been demonstrated in many studies, and highlight that acupuncture has been shown to be superior to conventional standard care. Patients will be told that, since acupuncture requires extensive training, the acupuncture therapists have received special training and are under close supervision of other team members so that they can provide a very high quality treatment. Patients will also receive the information that our team was very successful in using acupuncture to treat similar patients with CLBP, and we have shown the effectiveness of acupuncture in large clinical trials. Finally, patients will be informed that most patients are very satisfied with acupuncture treatment and would recommend it to other patients.

The booster visit at the second acupuncture session will begin with a short assessment of patients' experience of the first acupuncture session and any change in symptoms. In case of worsening or no change the patient will receive the information that this is a quite common experience of many patients and that the acupuncture effect will usually start after the second treatment and will increase over time. In case of improvement of health status the patient will receive the information that this initial response can be regarded as a positive sign for a strong ongoing response to acupuncture treatment. In both clinical situations, the effectiveness of acupuncture in treating patients with CLBP will be repeated again. In the written information, patients will be informed again about the effectiveness of acupuncture and encouraged to remember situations in which they can move well despite problems of pain. They will be encouraged to list on the sheet situations with reduced or without pain. Problem talk will be avoided and positive language about change in symptoms, pain perception, and optimism will be used.

8.2.2 Study Intervention B) Side Effects

The goal of the *intense safety briefing* is to provide information sensitizing patients to the presence of side effects during acupuncture treatment. The doctor will explain in detail the side effects that have been observed in a large study with over 2 million acupuncture treatments. Patients will receive the patient information leaflet that was developed based on this study and the doctor will advise them to read it carefully because he would like to discuss any questions they may have during the next visit. During the booster sessions the medical doctor will ask if side effects have occurred, explain the possible side effects again, and ask the patients if they have any questions about side effects.

8.2.3 Control Intervention A) Effectiveness

The *regular expectation briefing* it will be mentioned that acupuncture may help to overcome CLBP. However, since CLBP is a chronic condition, patients have to be realistic about treatment benefits. Only about half of the patients will benefit from acupuncture and professionals are unable to decide in advance if acupuncture will be beneficial for a specific patient. It will also be outlined and explained that sham acupuncture has a comparably high success rate. In the booster session, the medical doctor will do an assessment of back pain and limitations in daily life caused by back pain. The change of symptoms will be assessed: In case of worsening the patient will get the information that the patient might be a non-responder but he/she has to be patient. In case of an improvement the patient does not get any specific affirmation of progress but the doctor will note this improvement in the patient documentation. In the written material, the focus will be on pain perception and limitations. In contrast to the positive message (see above), the patient will be encouraged to focus on situations where pain is still present. The patient should describe situations according to the when?, what happened?, what did you do? matrix.

8.2.4 Control Intervention B) Side Effects

A *regular briefing about side effects* is part of the informed consent and will be delivered to all four groups. The regular safety briefing means that no additional information about side effects will be provided in the briefing sessions.

8.3 Compliance with Intervention

Regular appearance of patients to the acupuncture sessions is an indicator of compliance. Moreover, we will assess compliance of the briefing intervention and the acupuncture by reports of the professionals.

8.4 Data Collection and Follow-up for Withdrawn Participants

We will assess possible reasons for withdrawal or dropout at the last visit before dropout. A follow-up of withdrawn patients is not intended.

8.5 Concomitant Intervention(s)

Patients will be allowed to use oral non-steroidal anti-inflammatory drugs, if required (rescue medication). The use of corticosteroids or pain-relieving drugs that act through the central

nervous system, however, will be prohibited (specified list is in a studyspecific SOP). Any concomitant pain medication will be documented in the weekly diary (see document P6).

9 STUDY PROCEDURES

9.1 Study Flow Chart/Table of Study Procedures and Assessments

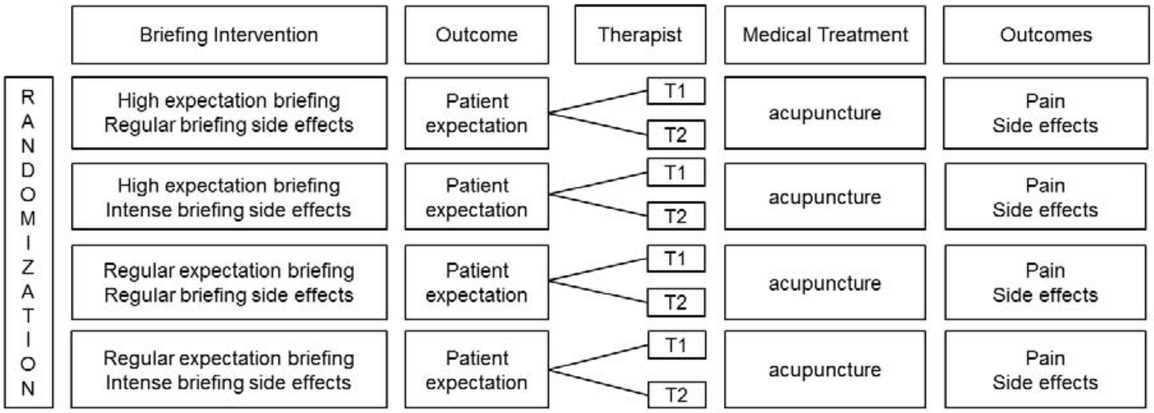

9.2 Assessments of Outcomes

9.2.1 Assessment of Primary Outcome

Patient expectations of acupuncture treatment (EAT) will be assessed by a self-report questionnaire. The main outcome measurement takes place at baseline, after the first face-to-face briefing intervention and after 2 weeks of treatment.

The back pain severity will be assessed by a numeric rating scale NRS asking for the average pain severity in the last 7 days (0-10) after 4 weeks of treatment.

For the side effects we will use a score that summarizes the side effects after the total duration of the treatment at 4 weeks: Before each acupuncture treatment session, side effects of the last treatment will be assessed and for each reported side effect patients will be asked to rate the intensity on a scale from 1-3 (low, moderate, strong). All intensity numbers will be summarized in the side effect score.

9.2.2 Assessment of Secondary Outcomes

Back pain severity: assessed as a secondary outcome by a numeric rating scale NRS (0-10) at 2 weeks (short term impact).

The back pain bothersomeness will be assessed by a numeric rating scale NRS asking for the average back pain bothersomeness in the last 7 days (0-10).

Self-reported health: PROMIS 29 (Patient-Reported Outcomes Measurement Information System) (Cella et al., 2010; Fries et al., 2011; Liu et al., 2010; Rothrock et al., 2010) is a well validated measure that was developed from the National Institutes of Health (NIH) in the USA

and is also available also in German. The PROMIS 29 is suggested as standard measure for CLBP studies by NIH (Deyo et al., 2014). Its profile covers the dimensions of anxiety, depression, fatigue, pain interference, physical function, sleep disturbance, and ability to participate in social roles and activities. All domains encompass four items with the exception of pain, which is measured by a single item. It will be measured at baseline, after 2 and 4 weeks of acupuncture and at the 6 months follow-up.

Working alliance with the acupuncturist: We will use 4 items of the Working Alliance Inventory (WAI-SR), (Wilmers et al., 2008) which measure the outcome on a 5 point scale. It will be measured after the first and the last acupuncture session.

We use the Perceived Sensitivity to Medicines Questionnaire (PSM), (Horne et al., 2013) to assess the belief to be influenced by medicines and experience side effects. It will be measured with 5 items on a 5 point Likert scale at baseline and at the end of the study.

Pain rescue medication (Brinkhaus et al., 2006): We will use a weekly diary to document the use of pain continuously. Patients will receive each week a new week-diary.

9.2.3 Additional measures:

We measure the optimism with the Life-orientation Test (LOT-R), (Glaesmer et al., 2008) at baseline.

Empathy of the physician: We will measure empathy using the German Version of the “Consultation and Relational Empathy“ (CARE) instrument (Neumann et al., 2008). The CARE is a one dimensional measure that consists of 9 items with a 5 point Likert scale. We will ask patients to complete it after the briefing and before the first acupuncture session.

Post-treatment guess: After completion of treatment, acupuncture therapists will be asked to guess which interventions patients received and their confidence with this guess. This will allow checking whether treatment providers were successfully blinded to briefing assignments.

Patients will be also asked to guess which briefing interventions they received. The patient guesses will be obtained after disclosure of the study aim at the end of the study (see ethical considerations), which might inform us about the success of the experimental manipulation.

9.2.4 Assessment of Safety Outcomes

9.2.4.1 Adverse Effects

We will measure adverse effects (= side effects of acupuncture treatment). See primary outcome (hypothesis 2).

9.2.4.2 Serious Adverse Events

Patients will be asked about serious adverse events by the acupuncturists (see documents A1 and A2). Moreover, patients will be advised to inform the study office if any serious unexpected event occurs. We will document this in the SAE report form.

9.2.5 Assessments in Participants who Prematurely Stop the Study

We will assess the causes for dropout by a questionnaire (document P11). We do not intend to have a follow-up of withdrawn patients.

9.3 Procedures at Each Visit

9.3.1 Screening

Screening will be done via telephone. The study center will clarify the basic inclusion criteria (see S1: demographic data, back pain duration and severity (≥ 4 NRS), acupuncture treatment experience, known pregnancy as reported by the patientIn case the patient is interested fulfills the inclusion criteria and does not fulfill any exclusion criteria, the informed consent form for Study Phase 1 (S4, which includes the informed consent only for the baseline questionnaire data) and the baseline questionnaire (P1) will be mailed to the patient. The patient information will be collected on a sheet (S5), which will be saved separately from other documents in a locked container with defined access.

The patient will be asked to read the documents, sign the informed consent and bring the signed consent together with the completed baseline questionnaire to the first visit with the physician. All visits will be scheduled with the patient and documented on S3.

9.3.2 Baseline (Visit 1a)

The first visit will take place at the Institute of Complementary and Integrative Medicine. The patient will be asked to hand in the informed consent for study phase 1 he had signed after the telephone interview and the baseline questionnaire to the study center. The study center will check them for completeness.
From the physician, the patient will receive the patient information for study phase 2 (B2) and will be asked to read it carefully.

Thereafter, the physician will inform the patient in detail about the study. The patient will have the opportunity to ask questions concerning the second phase of the study. The second informed consent will be given to the patient and he will be asked to read and sign it if he or she is still interested to participate in the study.

The physician will then check the inclusion criteria (See B1: demographic data, pain duration and severity (≥ 4 NRS), health status and patients' medical history/comorbidity).
The appointments fixed at the first phone screening will be provided to the physician by the study center. The physician and the patient will go through all the appointments to confirm that the patient can come for acupuncture on all dates.

In case of inclusion, the physician will assign the patient to a group allocation via secuTrial.
Afterwards, the briefing will take place for around 30 minutes. In the course of this briefing, material will be provided to the patient to take home (see brochures).
In addition the physician will give the patient the EAT (P2) and CARE (P4) and ask him to complete them immediately and to hand out P2 and to put P4 into an envelope. The physician will meanwhile fill in the adherence questionnaire B4.

9.3.3 First acupuncture (Visit 1b)

After the briefing, the patient will receive the first acupuncture.
After the acupuncture, the rescue medication weekly diary (P6) for week 1 will be handed out and explained to the patient. At the end, the acupuncturist will fill out the adherence questionnaire A1, the patient the acupuncturist's alliance (P5).

9.3.4 Visit 2

The second visit should take place within 2-7 days after the first acupuncture session. Before the second visit, participants will experience their booster session (according to the group allocation), which will take ca. 15 minutes (10 minutes effectiveness briefing and 5 minutes

safety briefing) and will be guided by the physician who also did the initial briefing session. The physician will again fill out the adherence questionnaire B5.

At the beginning of the acupuncture session, the side effects (P9) of the last acupuncture session will be measured and the weekly rescue medication diary (P6) will be checked. Patients will then receive the second acupuncture treatment.
After the session, the acupuncturist will fill out the adherence questionnaire A2.

9.3.5 Visit 3

This will be the third acupuncture session, which should take place within 2-7 days after the second acupuncture session. At the beginning, the side effects (P9) will be measured and the rescue medication diary (P6) will be collected. The patient will receive a new sheet and will be told to continue the diary for week 2.
Patients will then receive the acupuncture treatment.
Thereafter, the acupuncturists will fill out the adherence questionnaire A2.

After this session, the participant will receive the email/letter booster according to the group allocation.

9.3.6 Visit 4

Session 4 will be in the mid of the acupuncture treatment phase and hence, EAT (P2), PROMIS (P3) and NRS (P7) will be assessed after this session in addition to the normal session flow.

9.3.7 Visit 5-8

Sessions 5 to 8 will be the final four acupuncture sessions, which take place within 2 weeks. Each time, side effects and rescue medication diary will be recorded or checked, respectively, before the acupuncture starts. After visit 6, the patients will again receive an email/letter booster as after visit 3. After the very last acupuncture session (visit 8), the acupuncturist will answer the post-treatment questionnaire (A3) and the patient will fill in the acupuncturist's alliance questionnaire (WAI-SR, P5). Moreover, pain (P7), PROMIS (P3) and PSM (P8) will be assessed.

9.3.8 Follow up (Visit 9)

Six months after the last visit, patients will again be contacted and asked to fill out the follow up questionnaire (P12) including NRS (P7) and PROMIS (P3). They will receive the questionnaire by mail and will be asked to send it back to the study office in the prepaid questionnaire.

9.3.9 Study end (Visit 10)

The patient will be informed about the study aim and the patient information and the informed consent for study phase 3 will be handed out (B6). The patient can then withdraw his or her informed consent or sign the third consent form. The patient will be asked for a post-treatment guess about the group allocation (P10).

10 SAFETY

The Sponsor's SOPs provide more detail on safety reporting.

During the entire duration of the study, all serious adverse events (SAEs) that may be causally related to the study intervention are collected and documented in source documents. Reportable events are recorded in the case report form (CRF). Study duration encompassed the time from when the participant signs the informed consent until the last protocol-specific procedure has been completed, including a safety follow-up period.

In accordance with ClinO, serious adverse events (SAEs) are to be documented in a standardized manner and reported to the CEC if it cannot be excluded that the events are attributable to the intervention under investigation.

Furthermore, we will document adverse effects (= side effects of acupuncture treatment) (primary outcome for hypothesis 2) that might occur after each acupuncture session (assessment before the next acupuncture session).

10.1 Definitions

Adverse Effects (= side effects)

All unintended responses to a medicinal procedure related to any dose should be considered adverse effects. The phrase "responses to a medicinal procedure" means that a causal relationship between a medicinal procedure and an adverse effect is at least a reasonable possibility, i.e., the relationship cannot be ruled out.
For acupuncture we will especially focus on those side effects:

- bleeding at the injection site during acupuncture
- Bruises at the injection site after acupuncture
- Pain during acupuncture
- Inflammation at the injection site
- Vegetative symptoms such as
    - dizziness
    - transpiration
    - tachycardia
    - nausea
    - blood pressure problems
    - fatigue
- reinforcement of back pain
- Other pain (e.g. headaches or local muscle pain)

Serious Adverse Event

A serious adverse event is defined as any event which:

- requires inpatient treatment not envisaged in the protocol or extends a current
- hospital stay;
- results in permanent or significant incapacity or disability;
- is life-threatening or results in death; or
- causes a congenital anomaly or birth defect.

10.2 Recording and Assessment of Serious Adverse Events

The investigator has the responsibility for SAE identification, documentation, and assessing the causal relationship study intervention.

All SAEs will be fully documented in the appropriate CRF for up to 4 weeks after the intervention. At the last visit, patients will be instructed to report any SAE that occur within the next 4 weeks. For each SAE, the investigator will provide the onset, duration, treatment required, outcome and action taken with regard to the study intervention.

The assessment by the investigator with regard to the study intervention relation is done according to the following definitions:

Unrelated	• The event started in no temporal relationship to the medical intervention applied and<br>• The event can be definitely explained by underlying diseases or other situations.
Related	• The event started in a plausible temporal relationship to the medical intervention applied and<br>• The event cannot be definitely explained by underlying diseases or other situations.

10.3 Reporting of Serious Adverse Events

If, in the course of a clinical trial, serious adverse events occur in participants in Switzerland, and it cannot be excluded that the events are attributable to the intervention under investigation, the investigator must report these events to the CEC **within 15 days**.

Safety and protective measures

If immediate safety and protective measures have to be taken during the conduct of this clinical trial, the investigator must notify the CEC of these measures, and of the circumstances necessitating them, **within 7 days**.

Annual Safety Report

All SAEs will be summed up in the **annual safety report (ASR)** and submitted to the CEC. ASR shall contain:

- A summary of events including severity and causal relationship to the intervention and on the safety of participants.
- The accompanying letter provided with the Annual Safety Report should contain a short summary of the status of the clinical trial in Switzerland (number of centers open/closed, number of patients recruited/recruitment closed, and number of SAEs).

10.4 Follow up of (Serious) Adverse Events

Participants terminating the study (either regularly or prematurely) with reported ongoing SAE will return for a final follow-up investigation. This visit will take place up to 30 days after terminating the treatment period. Follow-up information on the outcome will be recorded on the respective SAE page in the CRF.

Follow-up investigations may also be necessary according to the investigator's medical judgment even if the participant has no SAE at the end of the study. However, information related to these investigations does not have to be documented in the CRF but must be noted in the source documents.

11 STATISTICAL METHODS

11.1 Hypotheses

1) Effectiveness
We will test the null hypothesis:
(1a) high expectation briefing = regular expectation briefing for change in expectation
The alternative hypothesis is:
(1a) high expectation briefing ≠ regular expectation briefing for change in expectation

We will test the null hypothesis:
(1b) high expectation briefing = regular expectation briefing for back pain,
The alternative hypothesis is:
(1b) high expectation briefing ≠ regular expectation briefing for back pain

We use this hierarchical test procedure to control for multiple testing. In a first step, we will test if the briefing changes the expectation. Only if the null hypothesis 1a was rejected (high expectation briefing ≠ regular expectation briefing) we will test in a second step whether the briefing changes pain after treatment (hypotheses 1b), using the same alpha level.

2) Side Effects
We will test null hypothesis:
(2) high side effect briefing = regular side effect briefing for amount of side effects reported
The alternative hypothesis is:
(2) high side effect briefing ≠ regular side effect briefing for amount of side effects reported

11.2 Determination of Sample Size

For sample size calculation we used nQuery Advisor (version 7.0).
Research question 1a: No previous data on expectancy change were available for trial planning purposes. For this study, we assume that the high expectation briefing is clinically meaningful better than a regular expectancy briefing in changing expectation, with a standardized mean difference (SMD) 0.5.
Assuming a power of 80% and a two-sided alpha level of 5%, we calculated that a sample of 128 patients (64 high expectation briefing and 64 regular expectation briefing) would enable detection of a difference of SMD 0.5 for the expectancy briefing intervention. Assuming a dropout rate of approximately 15%, we sought to enroll 150 patients.
Research question 1b: With the above mentioned sample we will have a power of 56-65% (n=128-150) to detect a significant difference between high and regular expectation briefing on average pain severity after 4 weeks. This assumption is based on our previous study

(Linde et al., 2007) with response rates of 54% in the high expectation group and 39% in the low expectation group that resulted in an SMD of .38.

Research question 2: Variation of the outcomes was not known prior to the study. Thus, the sample size was determined using two-group Fisher's exact tests of equal proportions (for binary outcomes). We expected to have 50% of patients with side effects to calculate a side effect score in the safety briefing group and 10% in the group without intense safety briefing (Witt et al., 2009). It showed that with 64 patients in each study group such differences in outcome scores could be detected with at least 99% power at a .05 two-sided significance level. This power analysis was conservative and the actual power of the study will be higher since we will use a continuous score and not only a binary variable.

11.3 Planned Analyses

11.3.1 Datasets to be Analyzed, Analysis Populations

The analyses will be tested for the intention to treat population using all available data.

Group allocations (briefing interventions):

- High expectancy vs. regular briefing
- Intense side effects briefing vs. regular side effects briefing

Measurement time points (data acquisition during the experiment):

- Baseline
- After 2 weeks
- After 4 weeks
- 6-months follow-up
- Study end

11.3.2 Primary Analysis

The significance level will be set to α = .05. It will be tested two-sided.

For the first hypotheses (1a) we will use an analysis of covariance (ANCOVA) with the fixed effects briefing group and sex and the covariate baseline expectation to determine the difference on expectation (EAT score) before the first acupuncture treatment between both briefing interventions.

For the second hypotheses (1b) to determine the difference in pain (numeric rating scale) after 4 weeks of acupuncture treatment we will use ANCOVA with the fixed effects briefing group, sex, therapist and the covariates baseline pain and optimism.

We use this hierarchical test procedure to control for multiple testing. In a first step, we will test if the briefing changes the expectation. Only if the null hypothesis 1a was rejected (high expectation briefing ≠ regular expectation briefing) we will test in a second step whether the briefing changes pain after treatment (hypotheses 1b), using the same alpha level.

Sensitivity analyses for both analyses will include a.) adding various covariates to the model (e.g. age, duration of disease, depression, empathy), b.) changing the study populations (per-protocol), and c.) using different multiple imputation methods for missing data (hot deck and regression methods). Confidence intervals and p-values from sensitivity analyses are

considered explorative. The research question if briefing effects on pain are mediated by initial treatment expectations will be tested according to the framework suggested by Baron and Kenny (Baron & Kenny, 1986; Hayes, 2009) in a hierarchical regression analysis.

Research question 2: As primary analysis, the total side effect score after 8 sessions will be compared between safety briefing groups using zero-inflated and hurdle models for count data. This analysis will require to appropriately imputing intermittent missing side effect scores and dropouts. Alternatively, a longitudinal analysis of the side effect scores, available from up to 8 sessions, will be achieved through an appropriate model extension using patient-specific random effects. As no standard software is available for this, problem- specific implementation using Markov Chain Monte Carlo (MCMC) or INLA (www.r-inla.org) will be used. Analyses will be conducted after LPLV by the statistician, presumably in the third year of the project.

In addition to the above mentioned research questions as an exploratory analysis for the total sample of included patients independent of group allocation to briefings the pain will be predicted by patient expectation before treatment using regression analyses with baseline pain severity, age, and sex as covariates. For the outcome pain two time points are relevant: 1) pain after 4 acupuncture sessions for the short term impact of expectations; 2) pain after 8 acupuncture sessions for the total impact of expectations on treatment outcomes.

11.3.3 Secondary Analyses

All secondary outcomes will be analyzed in an explorative manner; confidence intervals and p-values from secondary analyses are considered as explorative.

11.3.4 Interim Analyses

Not intended.

11.3.5 Safety Analysis

Analysis of the SAE frequencies.

11.3.6 Deviation(s) from the Original Statistical Plan

We will develop a detailed statistical plan before data analysis.

11.4 Handling of Missing Data and Drop-Outs

The questionnaire data will be checked at each study assessment to reduce missing data. Methods how to handle missing data in the statistical analyses are described above.

12 ELIGIBILITY OF THE PROJECT SITE(S)

See external document “10 QualifikationPruefort_Version1 21.8.2015”

13 DATA QUALITY ASSURANCE AND CONTROL

The Sponsor-Investigator is implementing and maintaining quality assurance and quality control systems with written SOPs and Working Instructions to ensure that trials are conducted and data are generated, documented (record), and reported in compliance with the protocol, GCP, and applicable regulatory requirement(s).

13.1 DATA HANDLING AND RECORD KEEPING

The study will strictly follow the protocol. If any changes become necessary, they must be laid down in an amendment to the protocol. All amendments of the protocol must be signed by the Sponsor-Investigator and if essential submitted to CEC.

13.1.1 Case Report Forms

The investigators will use paper case report forms (CRF), one for each enrolled study participant, to be filled in with all relevant data pertaining to the participant during the study. All participants who either entered the study or were considered not-eligible or were eligible but not enrolled into the study additionally have to be documented on a screening log. The investigator will document the participation of each study participant on the Screening- and Enrolment Log S2.

<u>Paper CRF:</u>

All requested information in the CRF should be completed in a neat legible manner. All corrections in a paper CRF must be made in a way that does not obscure the original entry. The correct data must be inserted, dated and initialed by the investigator. Data that are not available or not done should be made clear by adding NA or ND.

<u>Electronic CRF (eCRF):</u>

For data entry an internet-based secure data base RedCap® developed in agreement to the Good Clinical Practice (GCP) guidelines provided by the Clinical Trials Centre (CTC) Zurich will be used for this study (available from mid 2016). It is the responsibility of the investigator to assure that all data in the course of the study will be entered completely and correctly in the respective data base. Corrections in the eCRF may only be done by the investigator or by other authorized persons. In case of corrections the original data entries will be archived in the system and can be made visible. For all data entries and corrections date, time of day and person who is performing the entries will be generated automatically.

CRFs must be kept current to reflect participant status at each phase during the course of study. Participants must not to be identified in the CRF by name. Appropriate coded identification (e.g. Participant Number) must be used.

It must be assured that any authorized person, who may perform data entries and changes in the CRF, can be identified. A list with signatures and initials of all authorized persons will be filed in the study site file and the trial master file, respectively.

The investigators assure to perform a complete and accurate documentation of the participant data in the CRF. All data entered into the CRF must also be available in the individual participant file either as print-outs or as notes taken by either the investigator or another responsible person assigned by the investigator.

Essential documents must be retained for at least 10 years after the regular end or a premature termination of the respective study (KlinV Art. 45).

Any patient files and source data must be archived for the longest possible period of time according to the feasibility of the investigational site, e.g. hospital, institution or private practice.

13.1.2 Specification of Source Documents

The following documents are considered source data, including but not limited to:

- SAE worksheets
- Medical records from other department(s), or other hospital(s), or discharge letters and correspondence with other departments/hospitals, if participant visited any during the study period and the post-study period.
- CRFs

Source data must be available at the site to document the existence of the study participants and substantiate the integrity of study data collected. Source data must include the original documents relating to the study, as well as the medical treatment and medical history of the participant.

The following information (at least but not limited to) should be included in the source documents:

- Demographic data (age, sex)
- Inclusion and Exclusion Criteria details
- Participation in study and signed and dated Informed Consent Forms
- Visit dates
- Key efficacy and safety data (as specified in the protocol)
- SAEs (related)
- Reason for premature discontinuation
- Randomization number

13.1.3 Record Keeping / Archiving

All study data must be archived for a minimum of 10 years after study termination or premature termination of the clinical trial.
Electronic data will be kept on a secured data base on the USZ-Server. Other study documents will be archived in the archive of the Institute for Complementary and Integrative Medicine of the USZ.

13.2 Data Management

For analyses of the pseudonymous data from the CRFs, data will be entered into RedCap®, SPSS or Access data base which are stored on a secured server of the University Hospital Zurich. The University Hospital Zurich (USZ) has its own secured server that is only accessible by persons authorized by the institutional IT department. The regular backups as well as its quality control are carried out on the standard basis by the IT department.
According to Art. 9 ClinO, if consent is revoked, the health-related personal data of the person concerned will be anonymized after data evaluation has been completed.

13.3 Standard Operating Procedures and internal Monitoring

The trial will be conducted according to the Standard Operating Procedures (SOPs) of the Institute for Complementary and Integrative Medicine, University Hospital Zurich. The variables to be monitored will be determined in the SOPs.

Due to the low risk associated with our category A study, we will have an internal monitoring done by a researcher from the Institute of Complementary and Integrative Medicine, who is not

further involved in the study. Monitoring will take place at least three times. Before the beginning of the study, the following issues will be clarified: that the clinical investigation has to be conducted according to the study protocol, pursuance of responsibilities, and adherence to the study specific documentation according to the study protocol.
During each monitoring session, all or a part of the CRFs will be checked for completeness and validity, the frequency will be adapted to the study flow. If any points are unclear, these will be discussed with the trial researcher and, if necessary, with the study leader and coordinator. Adequateness of the facility will be monitored. The monitor will observe whether coworkers follow the valid study protocol and the safe and effective conduct and will decide about closure of the trial if significant risks are observed, or if the trial is unlikely to be concluded successful. The monitor must make sure that the written informed consent of each participant was given before participation.

13.4 Confidentiality, Data Protection

Direct access to source documents will be permitted for purposes of monitoring, audits and inspections during and after the study. The access to the study data is possible only through computers protected by password and for the study authorized persons. For the statistical analysis, only data coded by StudyID will be made available. The code-list that enables decoding of patients' identity will be secured by secuTrial® by the CTC (see Appendix).
The paper CRF including this data will be stored locked with tightly regulated access to the data.

14 PUBLICATION AND DISSEMINATION POLICY

After the statistical analysis of this trial the sponsor will make every endeavor to publish the data in a medical journal.

15 FUNDING AND SUPPORT

15.1 Funding

This study is an investigator initiated trial and funded by the Swiss National Foundation Grant number **105319_159833 / 1**.

16 INSURANCE

Insurance is covered by "Versicherung für klinische Versuche und nichtklinische Versuche" by Zürich Versicherungs-Gesellschaft AG (Policy no.: 14.970.888).

Any damage developed in relation to study participation is covered by this insurance. So as not to forfeit their insurance cover, the participants themselves must strictly follow the instructions of the study personnel. Participants must not be involved in any other medical treatment without permission of the principal investigator (emergency excluded). Medical emergency treatment

must be reported immediately to the investigator. The investigator must also be informed instantly, in the event of health problems or other damages during or after the course of study treatment.

The investigator will allow delegates of the insurance company to have access to the source data/documents as necessary to clarify a case of damage related to study participation. All involved parties will keep the patient data strictly confidential.

A copy of the insurance certificate will be placed in the Trial Master File.

17 REFERENCES

Baron, R. M. and Kenny, D. A. (1986), "The moderator–mediator variable distinction in social psychological research: Conceptual, strategic, and statistical considerations", *Journal of personality and social psychology,* Vol. 51 No. 6, pp. 1173.

Berman, B. M., Langevin, H. M., Witt, C. M. and Dubner, R. (2010), "Acupuncture for chronic low back pain", *New England Journal of Medicine,* Vol. 363 No. 5, pp. 454-461.

Bialosky, J. E., Bishop, M. D. and Cleland, J. A. (2010), "Individual expectation: an overlooked, but pertinent, factor in the treatment of individuals experiencing musculoskeletal pain", *Phys Ther,* Vol. 90 No. 9, pp. 1345-1355.

Bishop, F. L., Yardley, L., Prescott, P., Cooper, C., Little, P. and Lewith, G. T. (2014), "Psychological covariates of longitudinal changes in back-related disability in patients undergoing acupuncture".

Brinkhaus, B., Witt, C. M., Jena, S., Linde, K., Streng, A., Wagenpfeil, S., Irnich, D., Hummelsberger, J., Melchart, D. and Willich, S. N. (2003), "Acupuncture Randomized Trials (ART) in patients with chronic low back pain and osteoarthritis of the knee - design and protocols", *Forsch Komplementarmed Klass Naturheilkd,* Vol. 10 No. 4, pp. 185-191.

Brinkhaus, B., Witt, C. M., Jena, S., Linde, K., Streng, A., Wagenpfeil, S., Irnich, D., Walther, H. U., Melchart, D. and Willich, S. N. (2006), "Acupuncture in patients with chronic low back pain: A randomized controlled trial", *Archives of Internal Medicine,* Vol. 166 No. 4, pp. 450-457.

Cella, D., Riley, W., Stone, A., Rothrock, N., Reeve, B., Yount, S., Amtmann, D., Bode, R., Buysse, D. and Choi, S. (2010), "Initial adult health item banks and first wave testing of the Patient-Reported Outcomes Measurement Information System (PROMIS™) Network: 2005–2008", *Journal of Clinical Epidemiology,* Vol. 63 No. 11, pp. 1179.

Crow, R., Gage, H., Hampson, S., Hart, J., Kimber, A. and Thomas, H. (1999), "The role of expectancies in the placebo effect and their use in the delivery of health care: a systematic review", *Health Technol Assess,* Vol. 3 No. 3, pp. 1-96.

Deglon-Fischer, A., Barth, J. and Ausfeld-Hafter, B. (2009), "Complementary and alternative medicine in primary care in Switzerland", *Forschende Komplementaermedizin,* Vol. 16 No. 4, pp. 251-255.

Dellmann, T. and Lushington, K. (2012), "How natural therapists enhance positive expectations of patients", *Complementary therapies in clinical practice,* Vol. 18 No. 2, pp. 99-105.

Deyo, R. A., Dworkin, S. F., Amtmann, D., Andersson, G., Borenstein, D., Carragee, E., Carrino, J., Chou, R., Cook, K., DeLitto, A., Goertz, C., Khalsa, P., Loeser, J., Mackey, S., Panagis, J., Rainville, J., Tosteson, T., Turk, D., Von Korff, M. and Weiner, D. K. (2014), "Focus article report of the NIH task force on research standards for chronic low back pain", *Clin J Pain,* Vol. 30 No. 8, pp. 701-712.

Enck, P., Benedetti, F. and Schedlowski, M. (2008), "New insights into the placebo and nocebo responses", *Neuron,* Vol. 59 No. 2, pp. 195-206.

Fries, J., Rose, M. and Krishnan, E. (2011), "The PROMIS of better outcome assessment: responsiveness, floor and ceiling effects, and Internet administration", *The Journal of rheumatology,* Vol. 38 No. 8, pp. 1759-1764.
Glaesmer, H., Hoyer, J., Klotsche, J. and Herzberg, P. Y. (2008), "Die Deutsche Version des Life-Orientation-Tests (LOT-R) zum dispositionellen Optimismus und Pessimismus", *Zeitschrift für Gesundheitspsychologie,* Vol. 16 No. 1, pp. 26-31.
Haake, M., Mueller, H. H., Schade-Brittinger, C., Basler, H. D., Schaefer, H., Maier, C., Endres, H. G., Trampisch, H. J. and Molsberger, A. (2007), "German Acupuncture Trials (GERAC) for chronic low back pain: randomized, multicenter, blinded, parallel-group trial with 3 groups", *Archives of Internal Medicine,* Vol. 167 No. 17, pp. 1892-1898.
Hayes, A. F. (2009), "Beyond Baron and Kenny: Statistical mediation analysis in the new millennium", *Communication Monographs,* Vol. 76 No. 4, pp. 408-420.
Horne, R., Faasse, K., Cooper, V., Diefenbach, M. A., Leventhal, H., Leventhal, E. and Petrie, K. J. (2013), "The perceived sensitivity to medicines (PSM) scale: an evaluation of validity and reliability", *Br J Health Psychol,* Vol. 18 No. 1, pp. 18-30.
Hoy, D., Bain, C., Williams, G., March, L., Brooks, P., Blyth, F., Woolf, A., Vos, T. and Buchbinder, R. (2012), "A systematic review of the global prevalence of low back pain", *Arthritis Rheum,* Vol. 64 No. 6, pp. 2028-2037.
Iles, R. A., Davidson, M., Taylor, N. F. and O'Halloran, P. (2009), "Systematic review of the ability of recovery expectations to predict outcomes in non-chronic non-specific low back pain", *Journal of Occupational Rehabilitation,* Vol. 19 No. 1, pp. 25-40.
Kirsch, I. (1997), "Response expectancy theory and application: A decennial review", *Applied and preventive Psychology,* Vol. 6 No. 2, pp. 69-79.
Linde, K., Witt, C. M., Streng, A., Weidenhammer, W., Wagenpfeil, S., Brinkhaus, B., Willich, S. N. and Melchart, D. (2007), "The impact of patient expectations on outcomes in four randomized controlled trials of acupuncture in patients with chronic pain", *Pain,* Vol. 128 No. 3, pp. 264-271.
Liu, H., Cella, D., Gershon, R., Shen, J., Morales, L. S., Riley, W. and Hays, R. D. (2010), "Representativeness of the patient-reported outcomes measurement information system internet panel", *Journal of clinical epidemiology,* Vol. 63 No. 11, pp. 1169-1178.
MacPherson, H., Maschino, A. C., Lewith, G., Foster, N. E., Witt, C. M., Vickers, A. J. and Acupuncture Trialists, C. (2013), "Characteristics of acupuncture treatment associated with outcome: an individual patient meta-analysis of 17,922 patients with chronic pain in randomised controlled trials", *Public Library of Science One,* Vol. 8 No. 10, pp. e77438.
Mondloch, M. V., Cole, D. C. and Frank, J. W. (2001), "Does how you do depend on how you think you'll do? A systematic review of the evidence for a relation between patients' recovery expectations and health outcomes", *Canadian Medical Association Journal,* Vol. 165 No. 2, pp. 174-179.
Neumann, M., Wirtz, M., Bollschweiler, E., Warm, M., Wolf, J. and Pfaff, H. (2008), "Psychometric evaluation of the German version of the" Consultation and Relational Empathy"(CARE) measure at the example of cancer patients", *Psychotherapie, Psychosomatik, medizinische Psychologie,* Vol. 58 No. 1, pp. 5-15.
O'Connor, A. M., Pennie, R. A. and Dales, R. E. (1996), "Framing effects on expectations, decisions, and side effects experienced: the case of influenza immunization", *J Clin Epidemiol,* Vol. 49 No. 11, pp. 1271-1276.
Pach, D., Yang-Strobel, X., Ludtke, R., Roll, S., Icke, K., Brinkhaus, B. and Witt, C. M. (2013), "Standardized versus individualized ccupuncture for chronic low back pain: A randomized controlled trial", *Evid Based Complement Alternat Med,* Vol. 2013, pp. 125937.
Rothrock, N. E., Hays, R. D., Spritzer, K., Yount, S. E., Riley, W. and Cella, D. (2010), "Relative to the general US population, chronic diseases are associated with poorer health-related quality of life as measured by the Patient-Reported Outcomes Measurement Information System (PROMIS)", *Journal of clinical epidemiology,* Vol. 63 No. 11, pp. 1195-1204.

Schweizerischer Bundesrat. (2009), "Botschaft zum Bundesgesetz über die Forschung am Menschen", in, pp. 8045-8162.
Varelmann, D., Pancaro, C., Cappiello, E. C. and Camann, W. R. (2010), "Nocebo-induced hyperalgesia during local anesthetic injection", *Anesth Analg,* Vol. 110 No. 3, pp. 868-870.
Vickers, A. J., Cronin, A. M., Maschino, A. C., Lewith, G., MacPherson, H., Foster, N. E., Sherman, K. J., Witt, C. M., Linde, K. and Acupuncture Trialists Collaboration. (2012), "Acupuncture for chronic pain: individual patient data meta-analysis", *Arch Intern Med,* Vol. 172 No. 19, pp. 1444-1453.
Westra, H. A. and Dozois, D. J. A. (2006), "Preparing clients for cognitive behavioral therapy: A randomized pilot study of motivational interviewing for anxiety", *Cognitive Therapy and Research,* Vol. 30 No. 4, pp. 481-498.
Wilmers, F., Munder, T., Leonhart, R., Herzog, T., Plassmann, R., Barth, J. and Linster, H. W. (2008), "Die deutschsprachige Version des Working Alliance Inventory–short revised (WAI-SR). Ein schulenübergreifendes, ökonomisches und empirisch validiertes Instrument zur Erfassung der therapeutischen Allianz", *Klinische Diagnostik und Evaluation,* Vol. 1 No. 3, pp. 343-358.
Witt, C. M., Pach, D., Brinkhaus, B., Wruck, K., Tag, B., Mank, S. and Willich, S. N. (2009), "Safety of acupuncture: results of a prospective observational study with 229,230 patients and introduction of a medical information and consent form", *Forschende Komplementaermedizin,* Vol. 16 No. 2, pp. 91-97.

18 APPENDICES

1. Data management agreement for secuTrial® with CTC
