# Supplementary material for: Effect of Briefing on Acupuncture Treatment Outcome Expectations, Pain, and Adverse Side Effects Among Patients With Chronic Low Back Pain: A Randomized Clinical Trial
Source: JAMA Netw Open. 2021 Sep 10;4(9):e2121418. doi: 10.1001/jamanetworkopen.2021.21418 (PMC8433606; doi:10.1001/jamanetworkopen.2021.21418)
Supplement: Supplement 2. — eAppendix 1. Sample Size Calculation eAppendix 2. Exclusion Criteria eAppendix 3. Content of Different Effectiveness Briefings Before Minimal Acupuncture Treatment, in the Booster Session (Verbal), and in Booster Emails eAppendix 4. Description of Acupuncture Treatment eAppendix 5. Acupuncture Regimen eAppendix 6. Data Analysis eAppendix 7. Type of Adverse Side Effects per Session, Stratified by Briefing [file jamanetwopen-e2121418-s002.pdf]

Supplemental Online Content

Barth J, Muff S, Kern A, et al. Effect of briefing on acupuncture treatment outcome expectations, pain, and adverse effects among patients with chronic low back pain: a randomized clinical trial. *JAMA Netw Open.* 2021;4(9):e2121418. doi:10.1001/jamanetworkopen.2021.21418

eAppendix 1. Sample Size Calculation

eAppendix 2. Exclusion Criteria

eAppendix 3. Content of Different Effectiveness Briefings Before Minimal Acupuncture Treatment, in the Booster Session (Verbal), and in Booster Emails

eAppendix 4. Description of Acupuncture Treatment

eAppendix 5. Acupuncture Regimen

eAppendix 6. Data Analysis

eAppendix 7. Type of Adverse Side Effects per Session, Stratified by Briefing

This supplemental material has been provided by the authors to give readers additional information about their work.

© 2021 Barth J et al. *JAMA Network Open.*

eAppendix 1. Sample Size Calculation

For sample size calculation we used nQuery Advisor (version 7.0). Since no previous data on expectation change were available for trial planning purposes we assumed that the high expectation briefing is clinically meaningful better than a regular expectation briefing in changing expectation, with a standardized mean difference (SMD) 0.5. Assuming a power of 80% and a two-sided alpha level of 5%, we calculated that a sample of 128 patients (64 high expectation briefing and 64 regular expectation briefing) would enable detection of a difference of SMD 0.5 for the expectation briefing intervention. Assuming a dropout rate of approximately 15%, we sought to enroll 150 patients. Observational studies showed for the association of expectation and pain response rates of 54% in the high expectation group and 39% in the low expectation group which corresponds to a SMD of .38. Therefore, we had a power of 56 to 65% (n=128 to 150 patients) to detect a significant difference between high and regular expectation briefing on average pain intensity after 4 weeks. For the variation of the side effects no prior data using scores had been available.

© 2021 Barth J et al. *JAMA Network Open.*

eAppendix 2. Exclusion Criteria

Exclusion criteria were: acupuncture treatment within the last 12 months, breast-feeding or pregnant, blood coagulation disorder or took coagulation inhibiting drugs (exception aspirin), CLBP caused by a known malignant disease, trauma or a known rheumatic or autoimmune disorder, history or planned surgery of the spinal column of the low back in the next 6 months, regular intake of analgesics (> once per week) because of an additional disease, intake of centrally acting analgesics, regular intake of corticosteroids or injections with corticosteroids within the last 8 weeks before baseline, history of severe acute or chronic disorders that do not allow participation in the study, known alcohol or substance abuse, insufficient German language skills, current application for a pension claim.

© 2021 Barth J et al. *JAMA Network Open.*

eAppendix 3. Content of Different Effectiveness Briefings Before Minimal Acupuncture Treatment, in the Booster Session (Verbal), and in Booster Emails

	Regular expectation briefing	High expectation briefing
Topics of face-to-face communication		
Funding	"The study is financed by public funds. We hope that the study can provide new insights into the treatment of back pain."	"This study is financed by the Swiss National Fund. Our study concept was appraised as clinically relevant and important for developing new treatment options for pain patients."
Professional organizations	"The German Pain Society states: "In many cases, this needling therapy is as effective as typical western treatments. In general, acupuncture can heal what is dysfunctional but cannot repair disrupted tissues. To date, it remains partly unclear what	"The German Pain Society states: "Acupuncture, being an important treatment in Chinese Medicine, is recommended for many conditions by the World Health Organization (WHO) and leading Acupuncture Societies. It can improve pain in ligaments, muscles and joint capsules. Thanks to modern

© 2021 Barth J et al. *JAMA Network Open.*

	Regular expectation briefing	High expectation briefing
	exactly takes place in the body during acupuncture treatment.”	research investigations, the effectiveness of acupuncture can already be quite well explained.”
Patient organizations	“The Swiss Medical Society for Acupuncture, Chinese Medicine and Auriculomedicine mentions back pain on its list of indications.”	“According to the Rheumaliga Schweiz (Swiss Rheumatism League), acupuncture can decrease symptoms in non-inflammatory rheumatoid diseases remarkably.”
Expertise	“Our team works multidisciplinarily and has a lot of experience in integrative medicine. The therapists are experienced in chronic diseases and their treatment. Mrs. Prof. Witt is a renowned national and international expert in integrative medicine.”	“Our team, which is headed by Mrs. Prof. Witt, has a lot of expertise in acupuncture and consists of a lot of acupuncturists with long term experience. Mrs. Prof. Witt is a renowned national and international specialist for acupuncture in chronic pain conditions.”

© 2021 Barth J et al. *JAMA Network Open.*

	Regular expectation briefing	High expectation briefing
Specific training	"At our clinic, we also offer acupuncture. Some of our therapists were trained in acupuncture treatment. As you might know, acupuncture can be used as a treatment in different conditions. Our therapists are trained for different conditions, including back pain."	"Treating back pain with acupuncture is one main focus of our clinic. Our therapists are especially trained for acupuncture in chronic back pain. The training contained specific techniques, which are known to be very effective in the treatment of chronic back pain."
Other patients	"Every year, a lot of patients are treated at our clinic. We have a lot of experience in the treatment of chronic conditions."	"In our clinic, we have a lot of experience in treating patients with chronic back pain. Due to this, we can offer you a high-quality treatment."
Effectiveness	"Acupuncture can be beneficial in back pain. Both clinical trials and patients have reported this. It is possible that your pain also decreases. Obviously, not all patients do benefit from	"Acupuncture is a very beneficial treatment in back pain. This has been shown in both clinical trials and patient reports. You can expect noticeable pain relief. Most

© 2021 Barth J et al. *JAMA Network Open.*

	Regular expectation briefing	High expectation briefing
	acupuncture treatment. We know that around half of the patients show remarkable pain relief. This means the chance to profit or not is approximately 50%, like as if you would flip a coin. In other words, 100% of patients suffer from pain at the beginning of treatment. After acupuncture treatment, one half of patients is still in pain, and the other half shows pain relief."	patients show less pain after acupuncture treatment, and half show a highly noticeable pain reduction. This means you can expect a chance of 50% to have less back pain after the treatment. In other words, 100% of patients suffer from pain at the beginning of treatment. After acupuncture treatment, one half of the patients have a highly noticeable reduction in pain."
Long term effects	"One part of the patients can benefit from acupuncture over a longer period of time. This means you can still benefit from the acupuncture after the four weeks of treatment. This differs from usual drug medication, which requires a regular intake. As you	"Clinical trials show that the pain relief is persistent. This means that, even after the acupuncture treatment of four weeks, a remarkable persistent pain relief is very likely. This differs from usual drug medication, which requires a

© 2021 Barth J et al. *JAMA Network Open.*

	Regular expectation briefing	High expectation briefing
	can see in this graph (showing graph in brochure, see appendix), half of patients also show long term pain relief.	regular intake. As you can see in this graph (showing graph in brochure, see appendix), the same half of patients also show substantial pain relief in the long term.”
Topics of Booster 1		
Situations with pain	Patients were asked to list situations with a) little or less pain and b) a lot of pain. For both categories, they were asked to note what they did during each specific situation.	Patients were asked to list situations with little or less pain only. They were asked to note what they did during each specific situation.
Topics of Booster 2 (Email after 3 sessions)		
Effectiveness	“As you already know, acupuncture can be beneficial in back pain. Both clinical trials and	“As you already know, acupuncture is a very beneficial treatment for back

© 2021 Barth J et al. *JAMA Network Open.*

	Regular expectation briefing	High expectation briefing
	patients have reported this. It is possible that also your pain decreases.”	pain. This has been shown in both clinical trials and patient reports. You can expect noticeable pain relief.”
Pain reduction	“You already attended 3 acupuncture sessions and maybe your pain has already decreased a little. You will receive 5 further treatments, which might further relieve your pain.”	“You already attended 3 acupuncture sessions, and your pain has probably already decreased. You will receive 5 further treatments so that your back pain can further decrease.”
Topics of Booster 3 (Email after 6 sessions)		
Effectiveness and pain reduction	“As you already know, acupuncture can be beneficial for back pain. You already received 6 acupuncture treatments and maybe your pain has already relieved a little. You will receive two further	“As you already know, acupuncture is a very beneficial treatment for back pain. You will receive 2 further treatments. As we know from other research, that pain decreases after each

© 2021 Barth J et al. *JAMA Network Open.*

	Regular expectation briefing	High expectation briefing
	acupuncture sessions, which might further relieve your pain.	acupuncture session, and you can expect an ongoing positive effect of the treatment.”

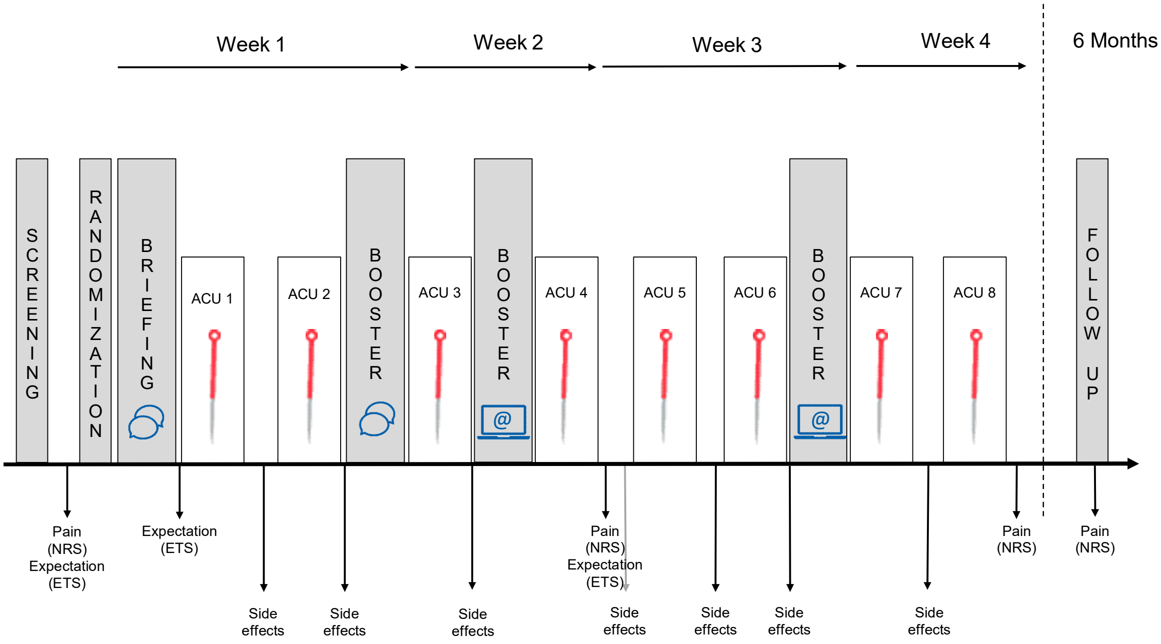

Treatment flow

© 2021 Barth J et al. *JAMA Network Open.*

eAppendix 4. Description of \Acupuncture Treatment

To reduce variance in the acupuncture outcomes, all patients received the same standardized minimal acupuncture treatment, which has been successfully used in a trial before and has been shown to be more effective than no treatment [26]. The treatment consisted of 8 treatment sessions of 45 minutes duration, applied over approximately four weeks (preferably two sessions per week). Fine needles (30 mm in length, Seirin B-Type) were superficially placed in six bilateral predefined points that are not traditional acupuncture points. In addition, they were slightly manually stimulated at the beginning and the end of the treatment session (see appendix for additional information). Needles stayed in the forearm for three minutes and in the remaining points for 25 minutes. We used points that were developed in a consensus procedure and were successfully implemented with beneficial effects on pain in previous trials [26 29]. Treatment was delivered by three treatment providers who were especially trained for this specific treatment and supervised by an MD acupuncturist. Patients were allowed to treat CLBP with non-steroidal anti-inflammatory drugs, if required. The use of pain medication had to be documented in a medication diary. All patients were informed that they received an acupuncture treatment that has been shown to be beneficial for CLBP.

© 2021 Barth J et al. *JAMA Network Open.*

Appendix 5. Acupuncture Regimen

Point	Location
MA point “Upper Arm”	In the middle of the insertion line of M. deltoideus (LI 14) and Acromion.
MA point “Forearm”	2 cun laterally (radial) of LU 3.
MA point “Back I”	1 cun ulnar of the proximal third of the line between Heart (HE) 3 and HE 7.
MA point “Back I”	5 cun laterally of the spine of lumbar vertebrum IV.
MA point “Back II”	5 cun laterally of the spine of lumbar vertebrum V.
MA point “Upper Leg III”	2 cun dorsally of GB 31 (avoidance of bladder meridian).

MA: Minimal Acupuncture

One cun is defined according to the rules of Traditional Chinese Medicine as the width of the interphalangeal joint of the patient’s thumb.

© 2021 Barth J et al. *JAMA Network Open.*

eAppendix 6. Data Analysis

The ETS score after the effectiveness briefing was analyzed using analysis of covariance (ANCOVA), with the expectation briefing group as the variable of interest adjusted for ETS baseline and gender as co-variates. If the result of the analysis indicated a significant effect of effectiveness briefing on the expectations, the analysis with pain intensity was considered as confirmatory otherwise as exploratory. The pain intensity after the minimal acupuncture treatment was analyzed using ANCOVA, with the expectation briefing as the variable of interest adjusted for pain intensity at the first visit, gender, treatment provider, and baseline optimism and pessimism. The adjusted means with 95% confidence intervals are reported for each treatment group (regular vs. high expectation briefing) for both outcomes. Missing data (multiple imputations with 50 iterations according to Rubin [35]) were imputed with the predictive mean matching method (pmm) in the case of continuous variables. For binary variables, a logistic regression model was used for the imputation, a multinomial logit model was used for factors with more than two levels, and an ordered logit model was used for ordered factors (>2 levels).

For the analysis of side effects, we compared the scores of each side effects briefing group (regular vs. intense) after session 1 to session 7. The side effect scores were treated as counts because they are expected to show a skewed distribution. Because each patient had up to 7 outcome values and because we expected overdispersion and zero-inflation, a longitudinal zero-inflated negative binomial model was used. This model has increased statistical power compared to a model with a total score as outcome, since it utilizes all outcomes of all patients. The model included the

© 2021 Barth J et al. *JAMA Network Open.*

side effect briefing group, gender, and time from study entry as explanatory variables, as well as patient-specific random intercepts. The model was fitted with the glmmTMB() function from the R package glmmTMB [36]. This longitudinal analysis allows for unbalanced missing data; thus, outcomes missing at random (MAR) are automatically accounted for and do not require any imputation (Ibrahim and Molenberghs, 2009). The results from the zero-inflated negative binomial regression were reported as the ratio of side effect scores (i.e., as the factor by which the side effect score changes from regular to intense side effects briefing) with 95% confidence intervals and p-values. In this setup, an estimated value of 1 corresponds to the null hypothesis that there is no effect of the briefing on the side effect scores. All data analyses were performed using R (Version 3.6.1) [37].

© 2021 Barth J et al. *JAMA Network Open.*

eAppendix 7. Type of Adverse Side Effects per Session, Stratified by Briefing

Values represent prevalence at each time point.

		After session 1	After session 2	After session 3	After session 4	After session 5	After session 6	After session 7
		n=149	n=145	n=142	n=141	n=138	n=136	n=132
Bleeding at puncture	Regular	4.1	12.9	15.9	11.8	8.8	13.4	13.6
	Intense	14.7	9.3	8.2	16.4	8.6	13.0	15.2
Haematoma at puncture	Regular	9.5	15.7	18.8	23.5	22.1	22.4	13.6
	Intense	21.3	21.3	24.7	27.4	28.6	27.5	18.2
Pain during acupuncture	Regular	47.3	48.6	55.1	58.8	51.5	46.3	45.5
	Intense	52.0	57.3	45.2	45.2	45.7	47.8	48.5
Inflammation at puncture	Regular	0.0	1.4	1.4	1.5	0.0	0.0	0.0
	Intense	0.0	1.3	2.7	2.7	4.3	4.3	4.6
Dizziness	Regular	9.5	12.9	7.2	7.4	10.3	6.0	6.1
	Intense	12.0	13.3	17.8	11.0	15.7	7.2	9.1
Sweating	Regular	9.5	12.9	14.5	8.8	7.4	6.0	10.6
	Intense	2.7	8.0	8.2	5.5	8.6	7.2	6.1
Heart palpitations	Regular	2.7	7.1	5.8	5.9	2.9	1.5	3.0
	Intense	0.0	1.3	4.1	2.7	5.7	5.8	7.6
Nausea	Regular	6.8	2.9	2.9	1.5	2.9	1.5	0.0

© 2021 Barth J et al. *JAMA Network Open.*

	Intense	6.7	6.7	9.6	1.4	5.7	4.3	6.1
Blood pressure problems	Regular	1.4	4.3	4.3	7.4	2.9	4.5	1.5
	Intense	2.7	4.0	2.7	5.5	11.4	4.3	9.1
Fatigue	Regular	21.6	25.7	21.7	22.1	23.9	21.2	15.2
	Intense	22.7	33.3	38.4	35.6	31.4	29.0	30.3
Increase in back pain	Regular	20.3	15.7	8.7	11.8	8.8	7.5	10.6
	Intense	22.7	17.3	17.8	20.5	20.0	14.5	13.6
Headache	Regular	12.2	14.3	8.7	11.8	7.4	9.0	3.0
	Intense	14.7	21.3	16.4	16.4	14.3	10.1	16.7
Muscular pain	Regular	23.0	12.9	10.1	16.2	13.2	13.4	13.6
	Intense	18.7	20.0	13.7	13.7	14.3	17.4	15.2

© 2021 Barth J et al. *JAMA Network Open.*
