# Supplementary material for: Effect of Briefing on Acupuncture Treatment Outcome Expectations, Pain, and Adverse Side Effects Among Patients With Chronic Low Back Pain: A Randomized Clinical Trial
Source: JAMA Netw Open. 2021 Sep 10;4(9):e2121418. doi: 10.1001/jamanetworkopen.2021.21418 (PMC8433606; doi:10.1001/jamanetworkopen.2021.21418)
Supplement: Supplement 3. — Data Sharing Statement [file jamanetwopen-e2121418-s003.pdf]

Data Sharing Statement

Barth. Effect of Briefing on Acupuncture Treatment Outcome Expectations, Pain, and Adverse Side Effects Among Patients With Chronic Low Back Pain. *JAMA Netw Open*. Published September 10, 2021.
doi:10.1001/jamanetworkopen.2021.21418

Data
Data available: No

Additional Information
Explanation for why data not available: Two of the authors are still preparing other manuscripts for secondary analyses of these data. Therefore, we can not share the data at this time. But we will make them accesible on reasonable request.
